# The FDA-approved drug nitazoxanide is a potent inhibitor of human seasonal coronaviruses acting at postentry level: effect on the viral spike glycoprotein

**DOI:** 10.3389/fmicb.2023.1206951

**Published:** 2023-08-29

**Authors:** Sara Piacentini, Anna Riccio, Silvia Santopolo, Silvia Pauciullo, Simone La Frazia, Antonio Rossi, Jean-Francois Rossignol, M. Gabriella Santoro

**Affiliations:** ^1^Department of Biology, University of Rome Tor Vergata, Rome, Italy; ^2^Institute of Translational Pharmacology, CNR, Rome, Italy; ^3^Romark Institute of Medical Research, Tampa, FL, United States

**Keywords:** antiviral, HCoV-229E, HCoV-OC43, HCoV-NL63, nitazoxanide, spike glycoprotein

## Abstract

*Coronaviridae* is recognized as one of the most rapidly evolving virus family as a consequence of the high genomic nucleotide substitution rates and recombination. The family comprises a large number of enveloped, positive-sense single-stranded RNA viruses, causing an array of diseases of varying severity in animals and humans. To date, seven human coronaviruses (HCoV) have been identified, namely HCoV-229E, HCoV-NL63, HCoV-OC43 and HCoV-HKU1, which are globally circulating in the human population (seasonal HCoV, sHCoV), and the highly pathogenic SARS-CoV, MERS-CoV and SARS-CoV-2. Seasonal HCoV are estimated to contribute to 15–30% of common cold cases in humans; although diseases are generally self-limiting, sHCoV can sometimes cause severe lower respiratory infections and life-threatening diseases in a subset of patients. No specific treatment is presently available for sHCoV infections. Herein we show that the anti-infective drug nitazoxanide has a potent antiviral activity against three human endemic coronaviruses, the Alpha-coronaviruses HCoV-229E and HCoV-NL63, and the Beta-coronavirus HCoV-OC43 in cell culture with IC_50_ ranging between 0.05 and 0.15 μg/mL and high selectivity indexes. We found that nitazoxanide does not affect HCoV adsorption, entry or uncoating, but acts at postentry level and interferes with the spike glycoprotein maturation, hampering its terminal glycosylation at an endoglycosidase H-sensitive stage. Altogether the results indicate that nitazoxanide, due to its broad-spectrum anti-coronavirus activity, may represent a readily available useful tool in the treatment of seasonal coronavirus infections.

## 1. Introduction

Coronaviruses (CoV), members of the family *Coronaviridae* (order *Nidovirales*), comprise a large number of enveloped, positive-sense single-stranded RNA viruses causing respiratory, enteric, hepatic and neurological diseases of varying severity in animals and humans (Cui et al., 2019; Fung and Liu, 2019). Coronaviruses have the largest identified RNA genomes (typically ranging from 27 to 32 kb) containing multiple open reading frames with an invariant gene order [a large replicase-transcriptase gene preceding structural (S-E-M-N) and accessory genes] (Fung and Liu, 2019). On the basis of their phylogenetic relationships and genomic structures, CoV are subdivided in four genera: Alpha-, Beta-, Gamma- and Delta-coronavirus; among these Alpha- and Beta-CoVs infect only mammals, whereas Gamma- and Delta-CoVs infect birds, and only occasionally can infect mammals (Cui et al., 2019). Human coronaviruses (HCoV) were discovered in the 1960s and were originally thought to cause only mild disease in humans (Cui et al., 2019; Fung and Liu, 2019). This view changed in 2002 with the SARS (Severe Acute Respiratory Syndrome) epidemic and in 2012 with the MERS (Middle East Respiratory Syndrome) outbreak, two zoonotic infections that resulted in mortality rates greater than 10 and 35%, respectively (Cui et al., 2019; Fung and Liu, 2019). Near the end of 2019, the seventh coronavirus known to infect humans, SARS-CoV-2, phylogenetically in the SARS-CoV clade, emerged in Wuhan, China. SARS-CoV-2 turned out to be a far more serious threat to public health than SARS-CoV and MERS-CoV because of its ability to spread more efficiently, making it difficult to contain worldwide with more than 768 million confirmed cases and over 6.9 million deaths reported worldwide, as of July 23rd, 2023.^1^ The clinical features of COVID-19, the disease associated with SARS-CoV-2, vary ranging from asymptomatic state to respiratory symptoms that, in a subset of patients, may progress to pneumonia, acute respiratory distress syndrome (ARDS), multi organ dysfunction and death (Tang et al., 2020; Lamers and Haagmans, 2022).

Only two HCoV, HCoV-OC43 and HCoV-229E, were known prior to the emergence of SARS-CoV (Tyrrell and Bynoe, 1965; Hamre and Procknow, 1966), while two more, HCoV-NL63 and HCoV-HKU1, were identified between 2004 and 2005 (van der Hoek et al., 2004; Woo et al., 2005). HCoV-OC43 and HCoV-HKU1 likely originated in rodents, while HCoV-229E and HCoV-NL63, similarly to SARS-CoV and MERS-CoV, originated in bats (Fung and Liu, 2019).

These four HCoV (seasonal HCoV, sHCoV) are globally distributed and are estimated to contribute to 15–30% of cases of common cold in humans (Lim et al., 2016; Liu et al., 2021). Although diseases are generally self-limiting, sHCoV can sometimes cause severe lower respiratory infections, including life-threatening pneumonia and bronchiolitis especially in infants, elderly people, or immunocompromised patients (Pene et al., 2003; Chiu et al., 2005; Gorse et al., 2009; Zhang et al., 2022); in addition, besides respiratory illnesses, sHCoV may cause enteric and neurological diseases (Arbour et al., 2000; Jacomy et al., 2006; Risku et al., 2010; Morfopoulou et al., 2016), while a possible involvement of HCoV-229E in the development of Kawasaki disease was suggested (Esper et al., 2005; Shirato et al., 2014).

**Figure 1:**
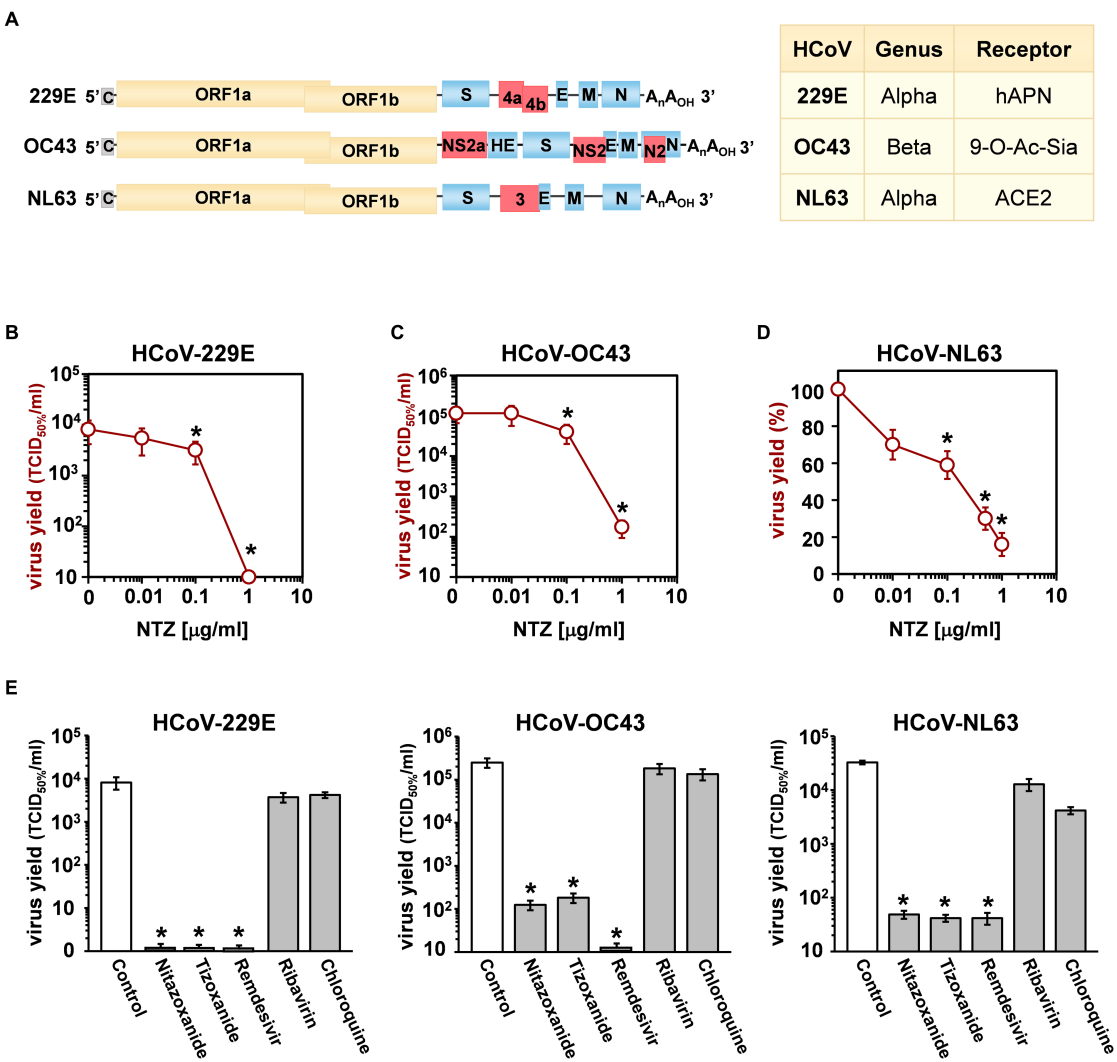
Antiviral activity of nitazoxanide against human seasonal coronaviruses. **(A)** Schematic representation of genome structure, classification and receptors of the human coronaviruses HCoV-229E, HCoV-OC43 and HCoV-NL63. ORF1a and ORF1b are represented as yellow boxes; genes encoding structural proteins spike (S), nucleocapsid (N), envelope (E), membrane (M), and hemagglutinin-esterase (HE) are shown as blue boxes, and genes encoding accessory proteins are shown as red boxes. hAPN, human aminopeptidase N; 9-O-Ac-Sia, N-acetyl-9-O-acetylneuraminic acid; ACE2, angiotensin-converting enzyme 2. **(B–D)** MRC-5 **(B,C)** and LLC-MK2 **(D)** cells mock-infected or infected with HCoV-229E **(B)**, HCoV-OC43 **(C)** or HCoV-NL63 **(D)** at an MOI of 0.1 TCID_50_/cell were treated with different concentrations of NTZ or vehicle immediately after the adsorption period. In the case of HCoV-NL63, NTZ was removed at 48 h after infection. Virus yield in cell supernatants was determined at 48 **(B)**, 96 **(C)** or 120 **(D)** hours p.i. by infectivity assay **(B,C)** or RNA quantification by qRT-PCR **(D)**. Data, expressed as TCID_50_/ml **(B,C)** or percent of untreated control **(D)**, represent the mean ± S.D. of duplicate samples. **(E)** MRC-5 cells infected with HCoV-229E or HCoV-OC43 and LLC-MK2 cells infected with HCoV-NL63 (0.1 TCID_50_/cell) were treated with 3 μM nitazoxanide, the NTZ active metabolite tizoxanide, remdesivir, chloroquine and ribavirin after virus adsorption. In the case of HCoV-NL63, NTZ and tizoxanide were removed at 48 h after infection. Virus yields were determined at 48 (HCoV-229E), 72 (HCoV-OC43) or 120 (HCoV-NL63) hours p.i. by infectivity assay. Data, expressed as TCID_50_/ml, represent the mean ± S.D. of duplicate samples. *p < 0.05; ANOVA test.

Whereas all seasonal coronaviruses cause respiratory tract infections, HCoV-OC43, HCoV-229E, HCoV-NL63 and HCoV-HKU1 are genetically dissimilar (Figure 1A), belonging to two distinct taxonomic genera (Alpha and Beta), and use different receptors that represent the major determinants of tissue tropism and host range (Fung and Liu, 2019). HCoV-229E and HCoV-NL63 have adopted cell surface enzymes as receptors, such as aminopeptidase N (APN) for HCoV-229E and angiotensin converting enzyme 2 (ACE2) for HCoV-NL63, while HCoV-OC43 and HCoV-HKU1 use 9-*O*-acetylated sialic acid as a receptor (Lim et al., 2016; Fung and Liu, 2019). In all cases, sHCoV infection is initiated by the binding of the spike (S) glycoprotein, anchored into the viral envelope, to the host receptor (Fung and Liu, 2019).

The coronavirus spike protein is a trimeric class-I fusion glycoprotein (Li, 2016); each monomer, with a molecular weight of 150–200 kDa after N-linked glycosylation (Lim et al., 2016), is synthesized as a fusogenically-inactive precursor that assembles into an inactive homotrimer, which is endoproteolytically cleaved by cellular proteases giving rise to a metastable complex of two functional subunits: S1 (bulb) containing the receptor-binding domain responsible for recognition and attachment to the host receptor, and the membrane-anchored S2 (stalk) that contains the fusion machinery (Li, 2016; Santopolo et al., 2021a). During synthesis in the infected cell, the nascent spike is imported into the endoplasmic reticulum (ER), where the protein is glycosylated (Sicari et al., 2020; Santopolo et al., 2021a). S glycoproteins passing the quality control mechanisms of the ER are transported to the ER/Golgi intermediate compartment (ERGIC), the presumed site of viral budding (Li, 2016; Fung and Liu, 2019).

Despite the fact that endemic seasonal coronaviruses may cause severe, life-threatening diseases in a subset of patients, no specific treatment is available for sHCoV infections.

Nitazoxanide, a thiazolide originally developed as an antiprotozoal agent and used in clinical practice for the treatment of infectious gastroenteritis (Rossignol et al., 2001, 2006b), and second-generation thiazolides have emerged as a new class of broad-spectrum antiviral drugs (Rossignol, 2014). Herein we investigated the antiviral activity of nitazoxanide against three human endemic coronaviruses belonging to two different genera: the Alpha-coronaviruses HCoV-229E and HCoV-NL63, and the Beta-coronavirus HCoV-OC43. We report that nitazoxanide is a potent inhibitor of HCoV replication acting at postentry level and interfering with Alpha- and Beta- sHCoV spike glycoprotein maturation.

## 2. Materials and methods

### 2.1. Cell culture and treatments

Human normal lung MRC-5 fibroblasts (American Type Culture Collection, ATCC, CCL-171) and rhesus monkey kidney LLC-MK2 cells (a kind gift from Lia van der Hoek, Academic Medical Center, University of Amsterdam) were grown at 37°C in a 5% CO_2_ atmosphere in minimal essential medium (MEM, Gibco 32360–026) for LLC-MK2 cells or EMEM (ATCC 30–2003) for MRC-5 cells, supplemented with 10% fetal calf serum (FCS), 2 mM glutamine and antibiotics. Nitazoxanide [2-acetyloxy-*N*-(5-nitro-2-thiazolyl) benzamide, Alinia] (NTZ) and tizoxanide (TIZ; Romark Laboratories, L.C.), remdesivir (MedChemExpress), ribavirin and chloroquine (Sigma-Aldrich), dissolved, respectively, in DMSO stock solution (NTZ, TIZ, remdesivir) or water (ribavirin, chloroquine), were diluted in culture medium, added to infected cells after the virus adsorption period, and maintained in the medium for the duration of the experiment, unless differently specified. Controls received equal amounts of DMSO vehicle, which did not affect cell viability or virus replication. Cell viability was determined by the 3-(4,5-dimethylthiazol-2-yl)-2,5-diphenyltetrazolium bromide (MTT) to MTT formazan conversion assay (Sigma-Aldrich), as described (Pizzato Scomazzon et al., 2019). The 50% lethal dose (LD_50_) was calculated using Prism 5.0 software (Graph-Pad Software Inc.). Microscopical examination of mock-infected or virus-infected cells was performed daily to detect virus-induced cytopathic effect and possible morphological changes and/or cytoprotection induced by the drug. Microscopy studies were performed using a Leica DM-IL microscope and images were captured on a Leica DC 300 camera using Leica Image-Manager500 software.

### 2.2. Coronavirus infection and titration

Human coronaviruses HCoV-229E (ATCC), HCoV-OC43 (ATCC) and HCoV-NL63 (strain Amsterdam-1, a kind gift from Lia van der Hoek, University of Amsterdam), were used for this study. Due to its poor ability to grow in cell culture the fourth known seasonal HCoV, HKU1, was not investigated. For HCoV-229E and HCoV-OC43 infection, confluent MRC-5 cell monolayers were infected for 1 h at 33°C at a multiplicity of infection (MOI) of 0.1 or 0.5 TCID_50_ (50% tissue culture infectious dose)/cell. For HCoV-NL63 infection, confluent LLC-MK2 cell monolayers were infected at 33°C for 2 h, as previously described (Milewska et al., 2014), at a MOI of 0.1 TCID_50_/cell. After the adsorption period, the viral inoculum was removed, and cell monolayers were washed three times with phosphate-buffered saline (PBS). Cells were maintained at 33°C in growth medium containing 2% FCS. For HCoV-229E and HCoV-OC43 infections, virus yield was determined in the supernatants collected from infected cells at different times after infection (p.i.) by TCID_50_ infectivity assay, as described previously (Santoro et al., 1988), using confluent MRC-5 cells in 96-well plates. In the case of HCoV-NL63, which requires 5 to 6 days for infectious progeny production and is characterized by a weak and transient cytopathic effect, virus yield was determined in the supernatant collected from infected cells at 120 h p.i. by viral RNA quantification, as previously reported (Castillo et al., 2022). Quantification of viral RNA in the supernatants of infected cells is described below. TCID_50_ infectivity assay in confluent LLC-MK2 cell monolayers in 96-well plates was also utilized in some experiments. The IC_50_ (50% inhibitory concentration) of the compounds tested was calculated using Prism 5.0 software.

### 2.3. HCoV RNA extraction and quantification

Measurement of sHCoV genomic RNA was performed by real-time quantitative reverse transcription-PCR (qRT-PCR), as described (Coccia et al., 2017). Briefly, total RNA from mock-infected or HCoV-infected cells was prepared using ReliaPrep RNA Cell Miniprep System (Promega) and reverse transcription was performed with PrimeScript RT Reagent Kit (Takara) in 20 μL final reaction volume (37°C for 15 min, 85°C for 5 s) according to the manufacturer’s protocol. Extracellular viral RNA was extracted from 200 μL of the supernatant of mock-infected or sHCoV-infected cell cultures with Viral Nucleic Acid Extraction Kit II (Geneaid) and 10 μL were subjected to reverse transcription using SuperScript™ VILO™ cDNA Synthesis Kit (Life Technologies) (25°C for 10 min, 42°C for 60 min and 85°C for 5 min), as described in the manufacturer’s protocol. Real-time PCR analysis was performed with CFX-96 (Bio-Rad) using SensiFAST SYBR® kit (Bioline) and primers specific for the membrane protein gene of HCoV-OC43 and HCoV-229E (Vijgen et al., 2005) or for the nucleoprotein gene of HCoV-NL63 (Pyrc et al., 2006). Relative quantities of selected mRNAs were normalized to ribosomal L34 RNA levels in the same sample. The sequences of the L34 primers were as follows: sense 5′-GGCCCTGCTGACATGTTTCTT-3′, antisense 5′-GTCCCGAACCCCTGGTAATAGA-3′. All reactions were made in triplicate using samples derived from three biological repeats with a negative control (mock-infected sample) and a “no template” control (NTC) included in each run. Subsequently the data were exported to Microsoft Excel and GraphPad Prism software for further analysis.

### 2.4. HCoV genomic RNA transfection

For sHCoV genomic RNA transfection experiments, MRC-5 cell monolayers were infected with HCoV-OC43 or HCoV-229E for 1 h at 33°C at an MOI of 0.1 TCID_50_/cell and sHCoV genomic RNA was extracted from the supernatants at 24 h p.i. using TRIzol-LS reagent (Life Technologies) as described in the manufacturer’s protocol. MRC-5 cell monolayers were mock-transfected or transfected with sHCoV genomic RNA (1 μg/mL) using TransIT-mRNA Transfection Kit (Mirus Bio) at 33°C. After 4 h, transfection medium was removed and cells were treated with NTZ or vehicle and maintained at 33°C in growth medium containing 2% FCS. At 24 h after treatment, culture supernatants were collected for virus progeny titer determination, and cell monolayers were processed for viral proteins detection.

### 2.5. Protein analysis, western blot and endoglycosidase digestion

For analysis of proteins whole-cell extracts (WCE) were prepared after lysis in High Salt Buffer (HSB; Santopolo et al., 2021b). Briefly, cells were washed twice with ice-cold PBS and then lysed in HSB (40 μL). After one cycle of freeze and thaw, and centrifugation at 16,000 ×*g* (10 min at 4°C), supernatant and pellet fractions were collected (Santoro et al., 1982). For Western blot analysis, cell extracts (20 μg/sample) were separated by SDS-PAGE under reducing conditions and blotted to a nitrocellulose membrane. Membranes were incubated with rabbit polyclonal anti-HCoV-OC43 spike (CSB-PA336163EA01HIY, Cusabio) or nucleocapsid (40643-T62, Sino Biological) antibodies, anti-HCoV-229E spike (PAB21477-100, LGC NAC Company) or nucleocapsid (40640-T62, Sino Biological) antibodies, anti β-actin (A2066, Sigma-Aldrich) antibodies, and monoclonal α-tubulin (T5168, Sigma-Aldrich) antibodies, followed by decoration with peroxidase-labeled anti-rabbit or anti-mouse IgG (Super-Signal detection kit, Pierce). Spike proteins densitometric and molecular weight analysis were performed with Image Lab Software 6.1, after acquisition on a ChemiDoc XRS+ (Bio-Rad).

For endoglycosidase digestion experiments, MRC-5 cells were mock-infected or infected with HCoV-OC43 or HCoV-229E at an MOI of 0.5 TCID_50_/cell and treated with NTZ (1 μg/mL) or vehicle immediately after the virus adsorption period. At 24 h p.i., cell monolayers were lysed in HSB buffer. After centrifugation at 16,000 ×*g* (10 min at 4°C), samples containing the same amount of protein (20 μg/sample) were processed for endoglycosidase-H (Endo-H, NEB) digestion using 5 milliunits Endo-H for 16 h at 37°C, according to the manufacturer’s protocol (Riccio et al., 2022). Digestions were terminated with the addition of Laemmli sample buffer (La Frazia et al., 2018). Samples were heated at 95°C for 5 min, separated by SDS-PAGE and processed for Western blot as described above. Quantitative evaluation of proteins was determined as described (Santoro et al., 1989; Amici et al., 2015). All results shown are representative of at least three independent experiments.

### 2.6. Immunofluorescence microscopy

MRC-5 cells infected with HCoV-OC43 or HCoV-229E were grown in 8-well chamber slides (Lab-Tek II) and, after the adsorption period, were treated with NTZ, or vehicle for 24 h. Cells were fixed, permeabilized and processed for immunofluorescence as described (Riccio et al., 2022) using anti-HCoV-OC43 or anti-HCoV-229E spike antibodies, followed by decoration with Alexa Fluor 555-conjugated antibodies (Molecular Probes, Invitrogen). Nuclei were stained with Hoechst 33342 (Molecular Probes, Invitrogen). Images were captured using a ZEISS Axio Observer Inverted Microscope and analyzed using ZEN 3.1 (blue edition) software. For confocal microscopy, images were acquired on Olympus FluoView FV-1000 confocal laser scanning system (Olympus America Inc., Center Valley, PA) and analyzed using Imaris (v6.2) software (Bitplane, Zurich, Switzerland). Images shown in all figures are representative of at least three random fields (scale-bars are indicated).

### 2.7. Statistical analysis

Statistical analyses were performed using Prism 5.0 software (GraphPad Software). Comparisons between two groups were made using Student’s *t*-test; comparisons among groups were performed by one-way ANOVA with Bonferroni adjustments. *p* ≤ 0.05 were considered significant. Data are expressed as the means ± standard deviations (SD) of results from duplicate or quadruplicate samples. Each experiment (in duplicate) was repeated at least twice.

## 3. Results

### 3.1. Antiviral activity of nitazoxanide against human seasonal coronaviruses

Three different human globally distributed coronaviruses, HCoV-229E, HCoV-OC43, and HCoV-NL63, were utilized for the current study. The genomic structure, classification, and receptors of these HCoVs are summarized in Figure 1A.

Nitazoxanide antiviral activity was first investigated in human lung MRC-5 and monkey kidney LLC-MK2 cells infected with HCoVs 229E (Figure 1B), OC43 (Figure 1C) and NL63 (Figure 1D) at the MOI of 0.1 TCID_50_/cell, and treated with different concentrations of the drug starting after the virus adsorption period. In the case of HCoV-NL63, which required 120 h for infectious progeny production, nitazoxanide was removed at 48 h after infection to avoid possible cytostatic/cytotoxic effects due to prolonged NTZ treatment *in vitro* (Wang et al., 2018). It should also be noted that, as previously reported in different cell lines (Loo et al., 2020), HCoV-OC43 and HCoV-229E display distinct replication kinetics, with HCoV-229E replicating more rapidly than HCoV-OC43, and viral loads peaking at 48 h and 96 h p.i. respectively under the conditions described.

At 48 (229E), 96 (OC43) and 120 (NL63) hours after infection, viral titers were determined in the supernatant of infected cells by TCID_50_ assay (Figures 1B,C) or viral RNA quantification (Figure 1D; Supplementary Figures S1A,B). Nitazoxanide showed a remarkable antiviral activity against all three HCoVs, reducing virus yield dose-dependently with IC_50_ values in the submicromolar range.

To compare the effect of nitazoxanide with other antiviral drugs, MRC-5 and LLC-MK2 cells were infected with the different HCoVs at the MOI of 0.1 TCID_50_/cell and treated with nitazoxanide, the NTZ bioactive metabolite tizoxanide, the antiviral drugs remdesivir and ribavirin, or chloroquine at the same concentration (3 μM) starting after virus adsorption. In the case of HCoV-NL63, NTZ and tizoxanide were removed at 48 h after infection. Virus yields were determined at 48 (HCoV-229E), 72 (HCoV-OC43) or 120 (HCoV-NL63) hours after infection by TCID_50_ assay. Nitazoxanide, tizoxanide and remdesivir showed comparable antiviral activity against all three HCoV, whereas chloroquine and ribavirin were not found to be effective when treatment was started after infection (Figure 1E).

**Figure 2:**
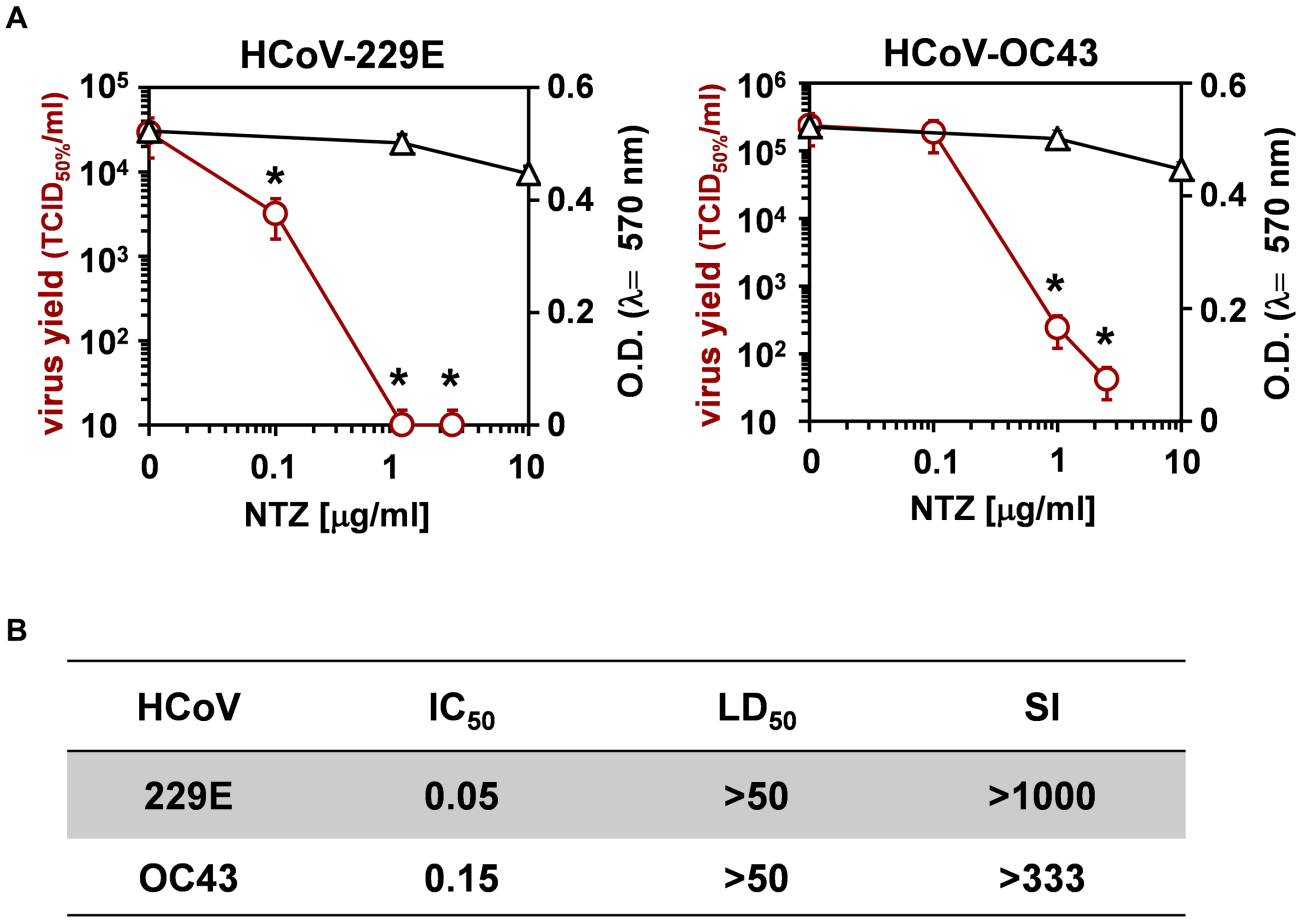
Effect of short treatment with nitazoxanide in OC43 and 229E HCoV-infected human lung cells. **(A,B)** MRC-5 cell monolayers mock-infected or infected with HCoV-229E and HCoV-OC43 (0.5 TCID_50_/cell) were treated with different concentrations of NTZ or vehicle immediately after the adsorption period. Virus yield (Ο, red line) was determined at 24 h p.i. by infectivity assay. In parallel, cell viability (△, black line) was determined by MTT assay in mock-infected cells **(A)**. Absorbance (O.D.) of converted dye was measured at λ = 570 nm. IC_50_ and LD_50_ in μg/ml **(B)** were calculated using Prism 5.0 software. Data represent the mean ± S.D. of duplicate samples. Selectivity indexes (SI) are indicated. *p < 0.05; ANOVA test.

Next, the effect of short-treatment with nitazoxanide was investigated in MRC-5 cells infected with HCoV-OC43 and HCoV-229E. MRC-5 cells were infected with the OC43 or 229E HCoV strains at the MOI of 0.5 TCID_50_/cell and treated with different concentrations of the drug starting after the virus adsorption period. At 24 h after infection, viral titers were determined in the supernatant of infected cells by TCID_50_ assay (Figure 2A) and viral RNA level quantification (Supplementary Figures S1C,D); in parallel, the effect of nitazoxanide on mock-infected cell viability was determined by MTT assay. The results, shown in Figures 2A,B, confirmed a potent antiviral activity of nitazoxanide, with IC_50_ values of 0.15 μg/mL and 0.05 μg/mL and selectivity indexes higher than 330 and 1,000 for HCoV-OC43 and HCoV-229E, respectively.

### 3.2. Nitazoxanide acts at postentry level

**Figure 3:**
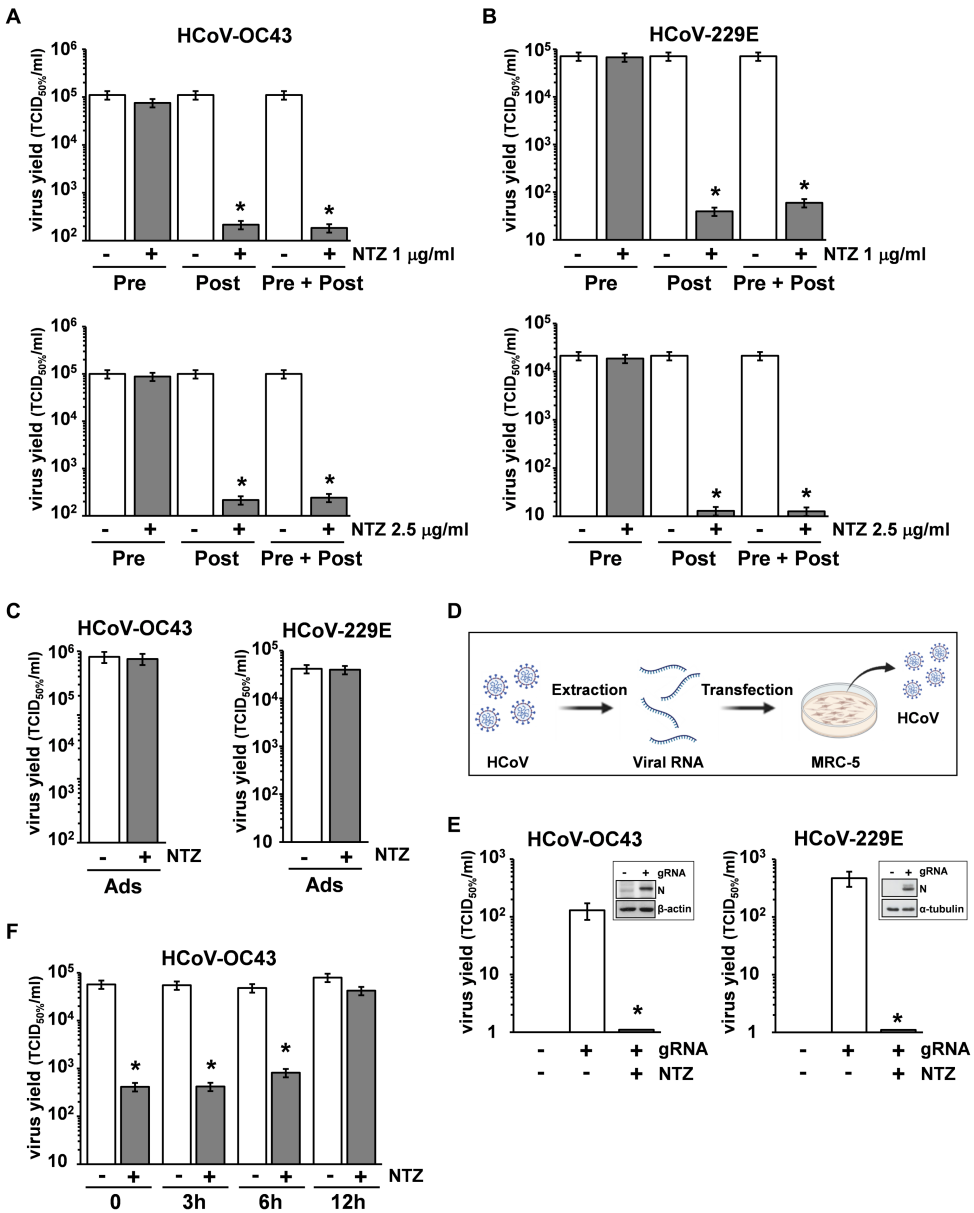
Nitazoxanide acts at postentry level. **(A,B)** MRC-5 cells mock-infected or infected with HCoV-OC43 **(A)** or HCoV-229E **(B)** (0.5 TCID_50_/cell) were treated with NTZ 1 μg/mL [**(A,B)** top; filled bars], NTZ 2.5 μg/mL [**(A,B)** bottom; filled bars] or vehicle (empty bars) 2 h before infection (Pre), after the adsorption period (Post), or 2 h before infection and treatment was continued during and after the adsorption period (Pre + Post). Virus yield was determined at 24 h p.i. by TCID_50_ infectivity assay. **(C)** MRC-5 cells mock-infected or infected with HCoV-OC43 or HCoV-229E were treated with NTZ (1 μg/mL) or vehicle only during the adsorption period (Ads). Virus yield was determined at 24 h p.i. by infectivity assay. **(A–C)** Data represent the mean ± S.D. of duplicate samples. *p < 0.01; Student’s *t*-test. **(D)** Schematic representation of HCoV genomic RNA transfection assay. **(E)** MRC-5 cells were transfected with HCoV-OC43 or HCoV-229E genomic RNA for 4 h and treated with NTZ (2.5 μg/mL) or vehicle for 24 h. Virus yield was determined at 24 h after treatment in the supernatant of transfected cells by TCID_50_ infectivity assay. Nucleocapsid (N) protein levels in HCoV-OC43 or HCoV-229E RNA-transfected cells are shown (insets). **(F)** MRC-5 cells infected with HCoV-OC43 were treated with NTZ (1 μg/mL) or vehicle at the indicated times after infection. Virus yield was determined at 24 h p.i. by infectivity assay. **(E,F)** Data represent the mean ± S.D. of duplicate samples. *p < 0.01; Student’s *t*-test.

To determine the effect of NTZ treatment before virus infection, MRC-5 cells were treated with NTZ (1 or 2.5 μg/mL) or vehicle for 2 h and the drug was removed before infection with OC43 or 229E HCoVs (0.5 TCID_50_/cell). In parallel, MRC-5 cells were infected with OC43 or 229E HCoVs (0.5 TCID_50_/cell) in the absence of the drug, and treated with 1 or 2.5 μg/mL NTZ or vehicle starting immediately after the adsorption period for the duration of the experiment. Alternatively, MRC-5 cells were treated 2 h before infection and treatment was continued during and after the adsorption period. Virus yield was determined at 24 h p.i. by TCID_50_ assay. As shown in Figures 3A,B, NTZ pretreatment did not significantly affect HCoV replication when the drug was removed before infection; on the other hand, treatment started after infection was effective in reducing infectious progeny production by approximately 3-fold in both HCoV models, without affecting cell viability (LD_50_ > 50 μg/mL).

In a different experiment, MRC-5 cells were infected with OC43 or 229E HCoV (0.5 TCID_50_/cell) and treated with NTZ (1 μg/mL) or vehicle only during the 1 h adsorption period, after which time the drug was removed. Virus yield was determined at 24 h p.i. by TCID_50_ assay. As shown in Figure 3C, NTZ treatment during virus adsorption did not affect HCoV replication. These results suggest that nitazoxanide acts at postentry level.

In order to rule out any effect of the drug on virus adsorption, entry or uncoating, OC43 and 229E HCoV genomic RNA was extracted and transfected into MRC-5 monolayers, as described in Materials and Methods, and schematically represented in Figure 3D. After 4 h, the transfection medium was removed and cells were treated with NTZ (2.5 μg/mL) or vehicle for 24 h. Virus yield was determined at 24 h after treatment in the supernatant of transfected cells. As shown in Figure 3E, genomic RNA transfection resulted in the production of infectious viral progeny (10^2^–10^3^ TCID_50_/ml) already at 24 h after transfection. Higher virus titers were detected at later times after transfection (data not shown). NTZ treatment greatly reduced the production of both HCoV-OC43 and HCoV-229E infectious particles after RNA transfection. Altogether, these results demonstrate that nitazoxanide is not acting on HCoV adsorption, entry or uncoating.

### 3.3. Nitazoxanide treatment impairs HCoV-OC43 and HCoV-229E spike maturation

Interestingly, NTZ-treatment initiated between 0 and 6 h p.i. was equally effective in inhibiting HCoV-OC43 virus replication, whereas the antiviral activity was impaired when treatment was started as late as 12 h p.i. (Figure 3F). Similar results were obtained after HCoV-229E infection (Supplementary Figure S2).

**Figure 4:**
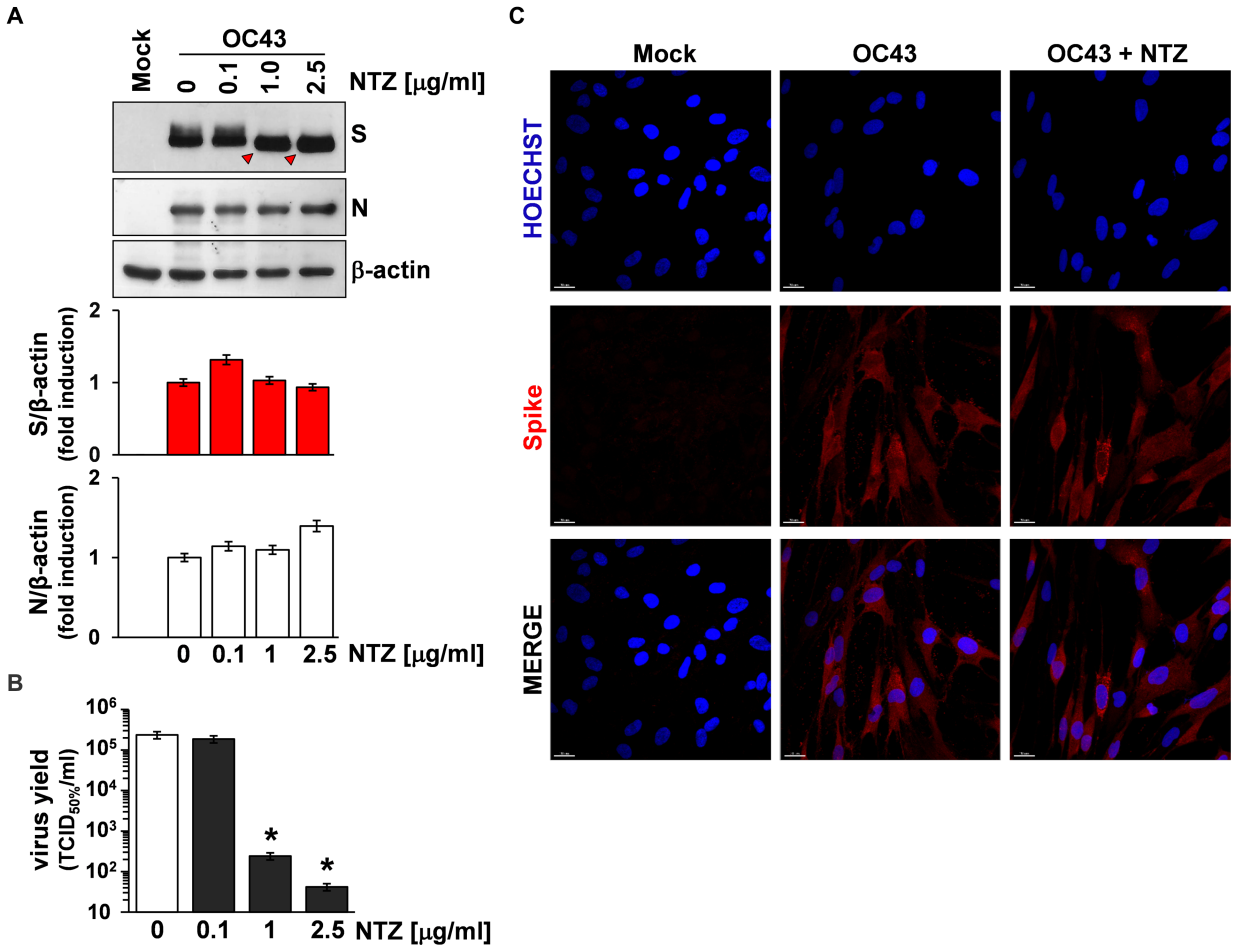
Effect of Nitazoxanide on HCoV-OC43 spike expression. **(A,B)** MRC-5 cells were mock-infected or infected with HCoV-OC43 at an MOI of 0.5 TCID_50_/cell and treated with different concentrations of NTZ or vehicle immediately after the virus adsorption period. After 24 h, whole cell extracts (WCE) were analyzed for levels of viral S and N proteins by IB using anti-spike and anti-N HCoV-OC43 antibodies [**(A)** top], and quantitated by scanning densitometry. Relative amounts of S and N proteins were determined after normalizing to β-actin [**(A)** bottom]. The faster-migrating form of the S protein in NTZ-treated cells is indicated by red arrowheads. In the same experiment virus yield was determined at 24 h p.i. in the supernatant of infected cells by TCID_50_ infectivity assay **(B)**. Data represent the mean ± S.D. of duplicate samples. *p < 0.01; ANOVA test. **(C)** Confocal images of HCoV-OC43 spike glycoprotein (red) in MRC-5 cells mock-infected or infected with HCoV-OC43 at an MOI of 0.5 TCID_50_/cell and treated with NTZ (1 μg/mL) or vehicle for 24 h. Nuclei are stained with Hoechst (blue). Merge images are shown. Scale bar, 20 μm.

**Figure 5:**
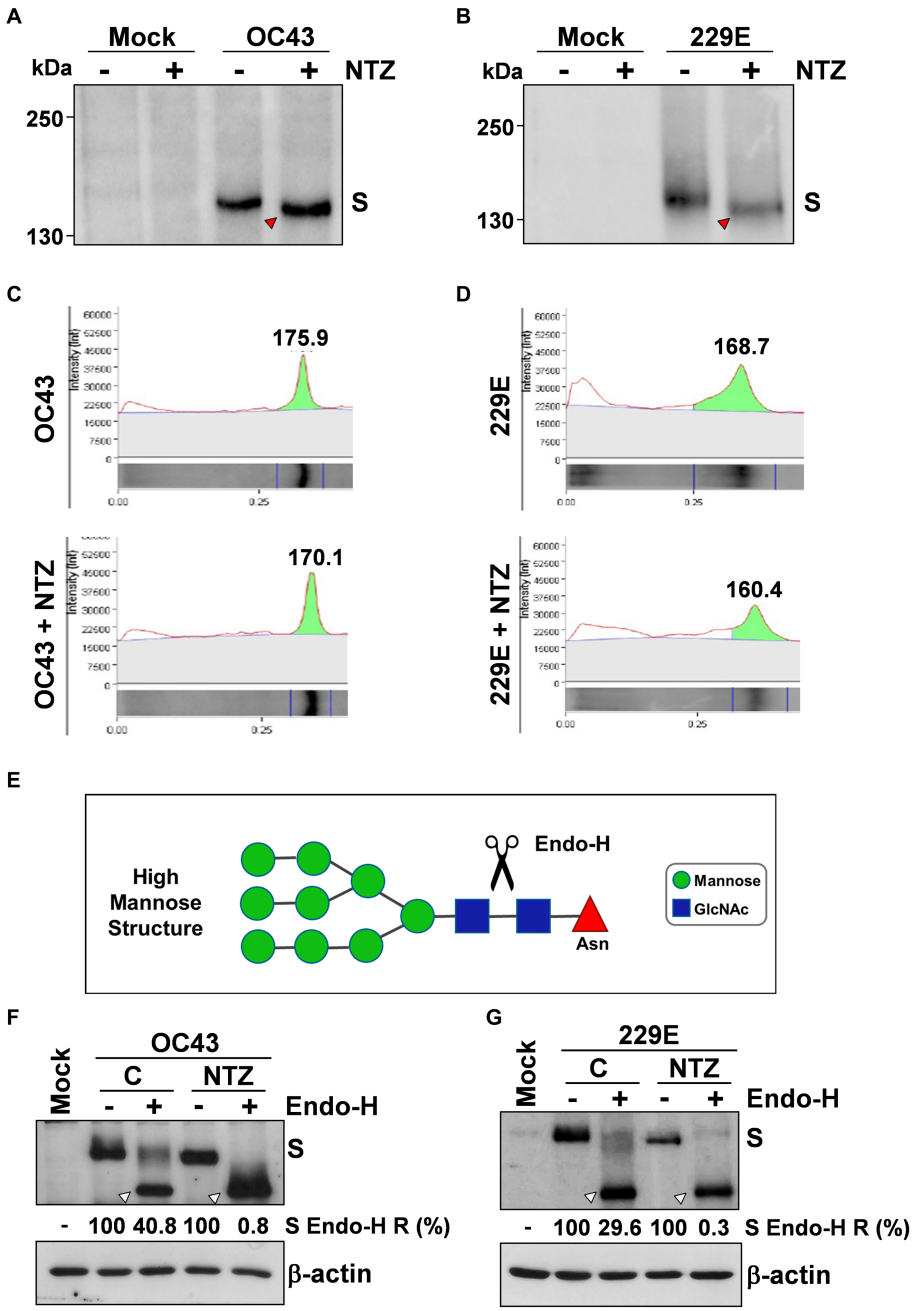
Nitazoxanide impairs HCoV-OC43 and HCoV-229E spike maturation at an Endo-H sensitive stage. **(A–D)** MRC-5 cells were mock-infected or infected with HCoV-OC43 **(A,C)** or HCoV-229E **(B,D)** at an MOI of 0.5 TCID_50_/cell and treated with NTZ (2.5 μg/mL) or vehicle immediately after the virus adsorption period. After 24 h, WCE were analyzed for levels of S protein by IB using anti-S HCoV-OC43 **(A,C)** or anti-S HCoV-229E **(B,D)** antibodies. Red arrowheads indicate the faster-migrating forms of the HCoV-OC43 **(A)** and HCoV-229E **(B)** spike proteins in NTZ-treated cells. Spike proteins densitometric and molecular weight analysis **(C,D)** are shown. (**E**) Diagram of substrate specificity of endoglycosidase-H (Endo-H). Mannose (green circles), *N*-acetylglucosamine (GlcNAc, blue squares) and asparagine (Asn, red triangle) residues are shown. Scissors represent the cleavage site of Endo-H. **(F,G)** MRC-5 cells were mock-infected or infected with HCoV-OC43 **(F)** or HCoV-229E **(G)** at an MOI of 0.5 TCID_50_/cell and treated with NTZ (1 μg/mL) or vehicle immediately after the virus adsorption period. After 24 h proteins were digested with Endo-H (+) or left untreated (−) and processed for IB analysis using anti-S HCoV-OC43 **(F)** or anti-S HCoV-229E **(G)** antibodies, and quantitated by scanning densitometry. The Endo-H-cleaved faster-migrating S forms are indicated by white arrowheads. The percentage of Endo-H-resistant (Endo-H R) S protein in the different samples is indicated.

To investigate whether NTZ may affect human coronavirus structural proteins expression, MRC-5 cells were infected with HCoV-OC43 (0.5 TCID_50_/cell) and treated with 0.1, 1 or 2.5 μg/mL NTZ or vehicle starting immediately after the adsorption period. At 24 h p.i., levels of the viral nucleocapsid (N) and spike (S) proteins were determined in the infected cells by immunoblot analysis using specific antibodies, and virus yield was determined in the culture supernatants by infectivity assay. As shown in Figure 4A, no significant differences in N and S protein levels were detected in NTZ-treated cells, as compared to control, at concentrations that caused a > 99% reduction in viral yield in the same samples (Figure 4B). Comparable levels of S-protein were also detected by confocal microscopy in MRC-5 cells infected with OC43 or 229E HCoVs (0.5 TCID_50_/cell) and treated with NTZ (1 μg/mL) or vehicle after the adsorption period for 24 h (Figure 4C; Supplementary Figure S3). Interestingly, treatment with NTZ at concentrations higher than 0.1 μg/mL caused an evident alteration in the electrophoretic mobility pattern of the spike glycoprotein (Figure 4A). An alteration in the molecular mass of the HCoV-OC43 spike protein of approximately 5.8 kDa was in fact detected in MRC-5 cells treated with 2.5 μg/mL NTZ (Figures 5A,C). In a parallel experiment, a similar alteration in the HCoV-229E spike molecular mass (8.3 kDa) was detected in MRC-5 cells treated with 2.5 μg/mL NTZ (Figures 5B,D), indicating an effect of the drug in the glycoprotein maturation process. These results are in line with our previous observation that the thiazolide affects the maturation of the SARS-CoV-2 S glycoprotein in human cells transfected with plasmids encoding different SARS-CoV-2 spike variants (Riccio et al., 2022). In this case, we have found that nitazoxanide blocks the maturation of the SARS-CoV-2 S glycoprotein at a stage preceding resistance to digestion by endoglycosidase-H (Endo-H), an enzyme that removes *N*-linked carbohydrate chains that have not been terminally glycosylated (Figure 5E; Ohuchi et al., 1997), thus impairing SARS-CoV-2 S intracellular trafficking and infectivity.

As indicated in the Introduction, glycosylation of coronavirus S proteins, like other cell surface glycoproteins, is initiated in the endoplasmic reticulum, adding the “high mannose” oligosaccharides; the mannose-rich sugar component is processed in the Golgi apparatus, and terminal glycosylation occurs in the *trans* cisternae of the Golgi apparatus (Xu and Ng, 2015).

To obtain insights on the effect of NTZ on HCoV S maturation, we therefore investigated whether nitazoxanide could affect HCoV-OC43 and HCoV-229E spike proteins terminal glycosylation. MRC-5 cells were infected with HCoV-OC43 or HCoV-229E (0.5 TCID_50_/cell) and treated with 1 μg/mL NTZ or vehicle starting immediately after the adsorption period. At 24 h p.i., protein aliquots (20 μg) from NTZ-treated or control cells were subjected to digestion with Endo-H and then analyzed by immunoblot. The results, shown in Figures 5F,G, indicate that, at this time, a fraction (approximately 40% in the case of HCoV-OC43, and 30% for HCoV-229E) of the spike proteins was found to be terminally glycosylated becoming Endo-H-resistant in control cells under the conditions described; notably, the spike proteins from NTZ-treated cells remained instead sensitive to digestion with the glycosidase up to 24 h after synthesis. Because acquisition of Endo-H resistance is a marker for transport into the *cis* and *middle* Golgi compartments (Ohuchi et al., 1997), these results indicate that nitazoxanide may impair S protein trafficking between the ER and the Golgi complex.

## 4. Discussion

*Coronaviridae* is recognized as one of the most rapidly evolving virus family as a consequence of the high genomic nucleotide substitution rates and recombination (Cui et al., 2019). As indicated in the Introduction, to date, seven human CoVs have been identified, namely HCoV-229E, HCoV-NL63, HCoV-OC43 and HCoV-HKU1, globally circulating in the human population, and the highly pathogenic SARS-CoV, MERS-CoV and SARS-CoV-2. Among these, SARS-CoV-2 is responsible for a devastating pandemic that is causing unprecedented public health interventions (COVID-19 Excess Mortality Collaborators, 2022). Given the proportion of the COVID-19 pandemic, major efforts have been directed over the past months towards a global vaccination plan.^2^ At the same time, the emergence of several SARS-CoV-2 spike variants that facilitate virus spread and may affect the efficacy of recently developed vaccines (Dong et al., 2021; Harvey et al., 2021; Planas et al., 2021; Carabelli et al., 2023), together with the short-lasting protective immunity typical of HCoV (Edridge et al., 2020), creates great concern and highlights the importance of identifying antiviral drugs to reduce coronavirus-related morbidity and mortality. So far, for SARS-CoV-2, two different RNA-dependent RNA polymerase (RdRp) inhibitors remdesivir (Santoro and Carafoli, 2020) and molnupiravir (Wahl et al., 2021; Jayk Bernal et al., 2022), and a viral protease inhibitor, Paxlovid (SARS-CoV-2 3CL protease inhibitor nirmatrelvir co-packaged with ritonavir), have been approved by health authorities in different countries (Hammond et al., 2022; Wen et al., 2022). On the other hand, no specific antiviral drug or vaccine are presently available for seasonal coronavirus infections.

Nitazoxanide has been proven to have a broad-spectrum antiviral activity (Rossignol, 2014; Li and De Clercq, 2020). In particular, nitazoxanide, its active metabolite tizoxanide and second generation thiazolides were found to be effective against several widespread RNA pathogens, including rotavirus, hepatitis C, and influenza and parainfluenza viruses in laboratory settings (Korba et al., 2008; Rossignol et al., 2009b; La Frazia et al., 2013, 2018; Belardo et al., 2015; Piacentini et al., 2018; Stachulski et al., 2021), as well as in clinical studies (Rossignol et al., 2006a, 2009a; Haffizulla et al., 2014). In the case of *Coronaviridae*, we first reported the effect of the drug against a canine strain of the virus (CCoV S-378) in canine A72 cells in 2007 (Santoro et al., 2007). Cao et al. (2015) showed that among 727 compounds tested against various strains of coronavirus, nitazoxanide was among the three most effective compounds tested (Cao et al., 2015). Nitazoxanide and tizoxanide were also found to be effective against MERS-CoV in LLC-MK2 cells with IC_50_s of 0.92 and 0.83 μg/mL, respectively, (Rossignol, 2016). As for SARS-CoV-2, at an early stage of the pandemic, Wang et al. reported that nitazoxanide inhibits SARS-CoV-2 replication in Vero E6 cells at low μM concentrations (EC_50_ = 2.12 μM; Wang et al., 2020); these observations were recently confirmed in different types of cells, including human lung-derived Calu-3 cells (Rocco et al., 2021; Son et al., 2022), as well as in animal models (Miorin et al., 2022). Tizoxanide was also recently found to be effective against SARS-CoV-2 in Vero E6 cells with an EC_50_ of 0.8 μg/mL (Riccio et al., 2022). More importantly several studies have recently shown an antiviral activity and clinical benefits of nitazoxanide in COVID-19 patients (Blum et al., 2021; Rocco et al., 2021, 2022; Silva et al., 2021; Rossignol et al., 2022a). It should be mentioned that no significant effect of nitazoxanide in the prevention or outcome of SARS-CoV-2 infections was reported in some studies (Fowotade et al., 2022; Sokhela et al., 2022). A recent study, while confirming the *in vitro* efficacy of nitazoxanide and tizoxanide against SARS-CoV-2, found that, in a SARS-CoV-2 virus challenge model in hamsters, oral and intranasal treatment with nitazoxanide failed to impair viral replication in affected organs, due to insufficient diffusion of the drug into the lungs and in the upper respiratory tract (Driouich et al., 2022), suggesting that the different results reported could depend on the dosage used, and that optimization of the NTZ formulation may be required to improve clinical efficacy of the drug.

In the case of seasonal HCoVs, it should be pointed out that treatment of subjects with laboratory-confirmed sHCoV infection with nitazoxanide administered orally twice daily for 5 days was associated with improvement in time to return to usual health and time until subjects are able to perform normal activities (Rossignol et al., unpublished results). However, the mechanism at the basis of the antiviral activity of nitazoxanide against coronaviruses is not yet understood.

We now show that nitazoxanide has a potent antiviral activity against three human endemic coronaviruses, the Alpha-coronaviruses HCoV-229E and HCoV-NL63, and the Beta-coronavirus HCoV-OC43 in cell culture with IC_50_ ranging between 0.05 and 0.15 μg/mL and high (>330) selectivity indexes. The fourth known seasonal HCoV, HKU1, was not investigated because of its poor ability to grow in cell culture.

We found that nitazoxanide does not affect virus adsorption, entry or uncoating of HCoVs, but acts at postentry level, interfering with the spike S-glycoprotein maturation at concentrations that do not inhibit S and N protein expression in the infected cell. These results confirm in two different actively-replicating HCoV models our previous observation that nitazoxanide affects the maturation of the SARS-CoV-2 S glycoprotein in human cells transfected with plasmids encoding different SARS-CoV-2 spike variants (Riccio et al., 2022). The results are also in line with our previous studies on influenza and parainfluenza viruses, where nitazoxanide was shown to impair terminal glycosylation and intracellular trafficking of the class-I viral fusion glycoproteins influenza hemagglutinin and paramyxovirus fusion proteins (Rossignol et al., 2009b; La Frazia et al., 2018; Piacentini et al., 2018).

As previously observed in the case of the hemagglutinin protein during human and avian influenza virus infection (Rossignol et al., 2009b; La Frazia et al., 2018), and of exogenously expressed SARS-CoV-2 S protein in human cells (Riccio et al., 2022), nitazoxanide was found to hamper the spike protein maturation at an Endo-H-sensitive stage, thus preventing its final processing. This effect has been previously associated to the drug-mediated inhibition of ERp57, an ER-resident glycoprotein-specific thiol-oxidoreductase which is essential for correct disulfide-bond architecture of selected viral proteins (Piacentini et al., 2018).

Because of the critical role of the spike protein in coronavirus assembly (Fung and Liu, 2019; Santopolo et al., 2021a), hampering S maturation may result in hindering progeny virus particle formation; however, we cannot exclude the existence of additional mechanisms that may contribute to the antiviral activity of thiazolides. Multiple mechanisms have in fact been implicated in the host-directed antiviral activity of nitazoxanide depending on the type of viral pathogen, including: interfering with the host cell energy metabolism and decreasing cellular ATP levels by mild uncoupling of mitochondrial oxidative phosphorylation (OXPHOS; Hammad et al., 2022; Rossignol et al., 2022b), and regulating the redox state of infected cells (Huang et al., 2023); induction of autophagy by inhibiting the Akt/mTOR/ULK1 signaling pathway in different types of cells (Lam et al., 2012; Shou et al., 2020); induction of PKR activation and subsequent phosphorylation of eIF2-α (Elazar et al., 2009; Ashiru et al., 2014); amplification of the host innate immune response, via an increase in RIG-I-like receptor activation, enhanced mitochondrial antiviral signaling protein and interferon regulatory factor 3 activities (Jasenosky et al., 2019); induction or enhancement of interferon (IFN)-stimulated gene expression (Gekonge et al., 2015; Petersen et al., 2016; Trabattoni et al., 2016; Jasenosky et al., 2019).

Interestingly, the antiviral activity of nitazoxanide and its bioactive metabolite tizoxanide against all sHCoV strains tested was found to be comparable to the direct-acting antiviral (DAA), potent RdRp inhibitor remdesivir (Santoro and Carafoli, 2020). It should be noted that, as in the case of other DAA, remdesivir may loose its efficacy due to the emergence of drug-resistant mutations in the target protein. This is in fact been already reported for use of remdesivir in SARS-CoV-2 infections *in vitro* after prolonged exposure to the drug, as well as in COVID-19 patients (Gandhi et al., 2022; Stevens et al., 2022). On the other hand nitazoxanide, being a host-directed antiviral (Rossignol, 2014; Li and De Clercq, 2020), it is unlikely to give rise to resistance.

In addition, remdesivir requires intravenous administration,^3^ whereas nitazoxanide is an oral drug with a well-established safety profile (Rossignol, 2014). Finally, due to their different mechanism of action, a possible use of combination of the two drugs could be hypothesized.

As indicated in the Introduction, HCoV-229E, HCoV-OC43 and HCoV-NL63, as well as HCoV-HKU1, are distributed globally, and generally cause mild upper respiratory tract diseases in adults; however, they may sometimes cause life-threatening diseases in a subset of patients (Arbour et al., 2000; Pene et al., 2003; Chiu et al., 2005; Jacomy et al., 2006; Gorse et al., 2009; Risku et al., 2010; Lim et al., 2016; Morfopoulou et al., 2016; Liu et al., 2021; Zhang et al., 2022). Interestingly, whereas HCoV-229E was suggested to be involved in the development of Kawasaki disease (Esper et al., 2005; Shirato et al., 2014), HCoV-OC43 has been shown to have neuroinvasive properties and to cause encephalitis in animal models (reviewed in Bergmann et al., 2006; Cheng et al., 2020). Moreover, both HCoV-OC43 and HCoV-229E were shown to establish persistent infections in cell cultures (Arbour et al., 1999a,b), while the presence of HCoV-OC43 RNA was detected in human brain autopsy samples from multiple sclerosis patients (Arbour et al., 2000; Cheng et al., 2020).

These observations, together with the knowledge that HCoV infection does not induce long-lasting protective immunity (Edridge et al., 2020), highlight the need for broad-spectrum anti-coronavirus drugs. The results described in the present study indicate that nitazoxanide, which has been used for decades in medical practice as a safe and effective antiprotozoal drug (Rossignol et al., 2001, 2006b; Rossignol, 2014), due to its broad-spectrum anti-coronavirus activity, may represent a readily available useful tool in the treatment of seasonal coronavirus infections.

## Data availability statement

The raw data supporting the conclusions of this article will be made available by the authors, without undue reservation.

## Ethics statement

Ethical approval was not required for the studies on humans in accordance with the local legislation and institutional requirements because only commercially available established cell lines were used.

## Author contributions

SPi, ARi, SS, and SPa performed the study on the antiviral activity and the transfection experiments. SPi, ARi, ARo, and SL performed the analysis of protein synthesis and maturation. MS and J-FR designed the study. MS, SPi, and ARi wrote the manuscript. All authors contributed to the article and approved the submitted version.

## Funding

This research was supported by Romark Laboratories LC, Tampa, Florida, and by a grant from the Italian Ministry of University and Scientific Research (PRIN project N 2010PHT9NF-006).

## Conflict of interest

Financial support for this study was in part provided by Romark Laboratories LC, the company that owns the intellectual property rights related to nitazoxanide. J-FR is an employee and stockholder of Romark Laboratories, LC.

The remaining authors declare that the research was conducted in the absence of any commercial or financial relationships that could be construed as a potential conflict of interest.

## Publisher’s note

All claims expressed in this article are solely those of the authors and do not necessarily represent those of their affiliated organizations, or those of the publisher, the editors and the reviewers. Any product that may be evaluated in this article, or claim that may be made by its manufacturer, is not guaranteed or endorsed by the publisher.

## Footnotes

^1^https://covid19.who.int/

^2^https://cdn.who.int/media/docs/default-source/immunization/covid-19/strategy-to-achieve-global-covid-19-vaccination-by-mid-2022.pdf?sfvrsn=5a68433c_5

^3^https://www.covid19treatmentguidelines.nih.gov/therapies/antivirals-including-antibody-products/remdesivir/

## References

[ref1] Amici C. La Frazia S. Brunelli C. Balsamo M. Angelini M. Santoro M. G. (2015). Inhibition of viral protein translation by indomethacin in vesicular stomatitis virus infection: role of eIF2α kinase PKR. Cell. Microbiol. 17, 1391–1404. doi: 10.1111/cmi.12446, PMID: 25856684 PMC7162271

[ref2] Arbour N. Côté G. Lachance C. Tardieu M. Cashman N. R. Talbot P. J. (1999a). Acute and persistent infection of human neural cell lines by human coronavirus OC43. J. Virol. 73, 3338–3350. doi: 10.1128/JVI.73.4.3338-3350.1999, PMID: 10074188 PMC104098

[ref3] Arbour N. Day R. Newcombe J. Talbot P. J. (2000). Neuroinvasion by human respiratory coronaviruses. J. Virol. 74, 8913–8921. doi: 10.1128/JVI.74.19.8913-8921.2000, PMID: 10982334 PMC102086

[ref4] Arbour N. Ekandé S. Côté G. Lachance C. Chagnon F. Tardieu M.. (1999b). Persistent infection of human oligodendrocytic and neuroglial cell lines by human coronavirus 229E. J. Virol. 73, 3326–3337. doi: 10.1128/JVI.73.4.3326-3337.1999, PMID: 10074187 PMC104097

[ref5] Ashiru O. Howe J. D. Butters T. D. (2014). Nitazoxanide, an antiviral thiazolide, depletes ATP-sensitive intracellular Ca(2+) stores. Virology 462-463, 135–148. doi: 10.1016/j.virol.2014.05.015, PMID: 24971706

[ref6] Belardo G. Cenciarelli O. La Frazia S. Rossignol J. F. Santoro M. G. (2015). Synergistic effect of nitazoxanide with neuraminidase inhibitors against influenza A viruses in vitro. Antimicrob. Agents Chemother. 59, 1061–1069. doi: 10.1128/AAC.03947-14, PMID: 25451059 PMC4335909

[ref7] Bergmann C. C. Lane T. E. Stohlman S. A. (2006). Coronavirus infection of the central nervous system: host-virus stand-off. Nat. Rev. Microbiol. 4, 121–132. doi: 10.1038/nrmicro1343, PMID: 16415928 PMC7096820

[ref8] Blum V. F. Cimerman S. Hunter J. R. Tierno P. Lacerda A. Soeiro A.. (2021). Nitazoxanide superiority to placebo to treat moderate COVID-19 – a pilot prove of concept randomized double-blind clinical trial. EClinicalMedicine 37:100981. doi: 10.1016/j.eclinm.2021.100981 34222847 PMC8235996

[ref9] Cao J. Forrest J. C. Zhang X. (2015). A screen of the NIH clinical collection small molecule library identifies potential anti-coronavirus drugs. Antivir. Res. 114, 1–10. doi: 10.1016/j.antiviral.2014.11.010, PMID: 25451075 PMC7113785

[ref10] Carabelli A. M. Peacock T. P. Thorne L. G. Harvey W. T. Hughes J. COVID-19 Genomics UK Consortium. (2023). SARS-CoV-2 variant biology: immune escape, transmission and fitness. Nat. Rev. Microbiol. 21, 162–177. doi: 10.1038/s41579-022-00841-7, PMID: 36653446 PMC9847462

[ref11] Castillo G. Mora-Díaz J. C. Nelli R. K. Giménez-Lirola L. G. (2022). Human air-liquid-interface organotypic airway cultures express significantly more ACE2 receptor protein and are more susceptible to HCoV-NL63 infection than monolayer cultures of primary respiratory epithelial cells. Microbiol. Spectr. 10:e0163922. doi: 10.1128/spectrum.01639-22, PMID: 35863002 PMC9431431

[ref12] Cheng Q. Yang Y. Gao J. (2020). Infectivity of human coronavirus in the brain. EBioMedicine 56:102799. doi: 10.1016/j.ebiom.2020.102799, PMID: 32474399 PMC7255711

[ref13] Chiu S. S. Hung Chan K. Wing Chu K. Kwan S. W. Guan Y. Man Poon L. L.. (2005). Human coronavirus NL63 infection and other coronavirus infections in children hospitalized with acute respiratory disease in Hong Kong, China. Rev. Infect. Dis. 40, 1721–1729. doi: 10.1086/430301, PMID: 15909257 PMC7107956

[ref14] Coccia M. Rossi A. Riccio A. Trotta E. Santoro M. G. (2017). Human NF-κB repressing factor acts as a stress-regulated switch for ribosomal RNA processing and nucleolar homeostasis surveillance. Proc. Natl. Acad. Sci. U. S. A. 114, 1045–1050. doi: 10.1073/pnas.1616112114, PMID: 28096332 PMC5293105

[ref15] COVID-19 Excess Mortality Collaborators (2022). Estimating excess mortality due to the COVID-19 pandemic: a systematic analysis of COVID-19-related mortality, 2020-21. Lancet 399, 1513–1536. doi: 10.1016/S0140-6736(21)02796-3, PMID: 35279232 PMC8912932

[ref16] Cui J. Li F. Shi Z. (2019). Origin and evolution of pathogenic coronaviruses. Nat. Rev. Microbiol. 17, 181–192. doi: 10.1038/s41579-018-0118-9, PMID: 30531947 PMC7097006

[ref17] Dong Y. Dai T. Wang B. Zhang L. Zeng L. H. Huang J.. (2021). The way of SARS-CoV-2 vaccine development: success and challenges. Signal Transduct. Target. Ther. 6:387. doi: 10.1038/s41392-021-00796-w, PMID: 34753918 PMC8575680

[ref18] Driouich J. S. Cochin M. Touret F. Petit P. R. Gilles M. Moureau G.. (2022). Pre-clinical evaluation of antiviral activity of nitazoxanide against SARS-CoV-2. EBioMedicine 82:104148. doi: 10.1016/j.ebiom.2022.104148, PMID: 35834886 PMC9271885

[ref19] Edridge A. W. D. Kaczorowska J. Hoste A. C. R. Bakker M. Klein M. Loens K.. (2020). Seasonal coronavirus protective immunity is short-lasting. Nat. Med. 26, 1691–1693. doi: 10.1038/s41591-020-1083-1, PMID: 32929268

[ref20] Elazar M. Liu M. McKenna S. A. Liu P. Gehrig E. A. Puglisi J. D.. (2009). The anti-hepatitis C agent nitazoxanide induces phosphorylation of eukaryotic initiation factor 2alpha via protein kinase activated by double-stranded RNA activation. Gastroenterology 137, 1827–1835. doi: 10.1053/j.gastro.2009.07.056, PMID: 19664635

[ref21] Esper F. Shapiro E. D. Weibel C. Ferguson D. Landry M. L. Kahn J. S. (2005). Association between a novel human coronavirus and Kawasaki disease. J. Infect. Dis. 191, 499–502. doi: 10.1086/428291, PMID: 15655771 PMC7199489

[ref22] Fowotade A. Bamidele F. Egbetola B. Fagbamigbe A. F. Adeagbo B. A. Adefuye B. O.. (2022). A randomized, open-label trial of combined nitazoxanide and atazanavir/ritonavir for mild to moderate COVID-19. Front. Med. 9:956123. doi: 10.3389/fmed.2022.956123, PMID: 36160134 PMC9493023

[ref23] Fung T. S. Liu D. X. (2019). Human coronavirus: host-pathogen interaction. Annu. Rev. Microbiol. 73, 529–557. doi: 10.1146/annurev-micro-020518-115759, PMID: 31226023

[ref24] Gandhi S. Klein J. Robertson A. J. Peña-Hernández M. A. Lin M. J. Roychoudhury P.. (2022). De novo emergence of a remdesivir resistance mutation during treatment of persistent SARS-CoV-2 infection in an immunocompromised patient: a case report. Nat. Commun. 13:1547. doi: 10.1038/s41467-022-29104-y, PMID: 35301314 PMC8930970

[ref25] Gekonge B. Bardin M. C. Montaner L. J. (2015). Short communication: Nitazoxanide inhibits HIV viral replication in monocyte-derived macrophages. AIDS Res. Hum. Retrovir. 31, 237–241. doi: 10.1089/aid.2014.0015, PMID: 25303025 PMC4313412

[ref26] Gorse G. J. O’Connor T. Z. Hall S. L. Vitale J. N. Nichol K. L. (2009). Human coronavirus and acute respiratory illness in older adults with chronic obstructive pulmonary disease. J. Infect. Dis. 199, 847–857. doi: 10.1086/597122, PMID: 19239338 PMC7110218

[ref27] Haffizulla J. Hartman A. Hoppers M. Resnick H. Samudrala S. Ginocchio C.. (2014). Effect of nitazoxanide in adults and adolescents with acute uncomplicated influenza: a double-blind, randomised, placebo-controlled, phase 2b/3 trial. Lancet Infect. Dis. 14, 609–618. doi: 10.1016/S1473-3099(14)70717-0, PMID: 24852376 PMC7164783

[ref28] Hammad N. Ransy C. Pinson B. Talmasson J. Bréchot C. Bouillaud F.. (2022). Antiviral effect of thiazolides relies on mitochondrial mild uncoupling. bioRxiv. doi: 10.1101/2022.09.16.508272

[ref29] Hammond J. Leister-Tebbe H. Gardner A. Abreu P. Bao W. Wisemandle W.. (2022). Oral nirmatrelvir for high-risk, nonhospitalized adults with Covid-19. N. Engl. J. Med. 386, 1397–1408. doi: 10.1056/NEJMoa2118542, PMID: 35172054 PMC8908851

[ref30] Hamre D. Procknow J. J. (1966). A new virus isolated from the human respiratory tract. Proc. Soc. Exp. Biol. Med. 121, 190–193. doi: 10.3181/00379727-121-30734, PMID: 4285768

[ref31] Harvey W. T. Carabelli A. M. Jackson B. Gupta R. K. Thomson E. C. Harrison E. M.. (2021). SARS-CoV-2 variants, spike mutations and immune escape. Nat. Rev. Microbiol. 19, 409–424. doi: 10.1038/s41579-021-00573-0, PMID: 34075212 PMC8167834

[ref32] Huang Z. Zheng H. Wang Y. Wang X. Wang C. Liu Y.. (2023). The modulation of metabolomics and antioxidant stress is involved in the effect of nitazoxanide against influenza a virus *in vitro*. Acta Virol. 67:11612. doi: 10.3389/av.2023.11612

[ref33] Jacomy H. Fragoso G. Almazan G. Mushynski W. E. Talbot P. J. (2006). Human coronavirus OC43 infection induces chronic encephalitis leading to disabilities in BALB/C mice. Virology 349, 335–346. doi: 10.1016/j.virol.2006.01.049, PMID: 16527322 PMC7111850

[ref34] Jasenosky L. D. Cadena C. Mire C. E. Borisevich V. Haridas V. Ranjbar S.. (2019). The FDA-approved oral drug nitazoxanide amplifies host antiviral responses and inhibits Ebola virus. iScience. 19, 1279–1290. doi: 10.1016/j.isci.2019.07.003, PMID: 31402258 PMC6831822

[ref35] Jayk Bernal A. Gomes da Silva M. M. Musungaie D. B. Kovalchuk E. Gonzalez A. Delos Reyes V.. (2022). Molnupiravir for oral treatment of Covid-19 in nonhospitalized patients. N. Engl. J. Med. 386, 509–520. doi: 10.1056/NEJMoa2116044, PMID: 34914868 PMC8693688

[ref36] Korba B. E. Montero A. B. Farrar K. Gaye K. Mukerjee S. Ayers M. S.. (2008). Nitazoxanide, tizoxanide and other thiazolides are potent inhibitors of hepatitis B virus and hepatitis C virus replication. Antivir. Res. 77, 56–63. doi: 10.1016/j.antiviral.2007.08.005, PMID: 17888524

[ref37] La Frazia S. Ciucci A. Arnoldi F. Coira M. Gianferretti P. Angelini M.. (2013). Thiazolides, a new class of antiviral agents effective against rotavirus infection, target viral morphogenesis, inhibiting viroplasm formation. J. Virol. 87, 11096–11106. doi: 10.1128/JVI.01213-13, PMID: 23926336 PMC3807293

[ref38] La Frazia S. Piacentini S. Riccio A. Rossignol J. F. Santoro M. G. (2018). The second-generation thiazolide haloxanide is a potent inhibitor of avian influenza virus replication. Antivir. Res. 157, 159–168. doi: 10.1016/j.antiviral.2018.06.008, PMID: 29908209

[ref39] Lam K. K. Zheng X. Forestieri R. Balgi A. D. Nodwell M. Vollett S.. (2012). Nitazoxanide stimulates autophagy and inhibits mTORC1 signaling and intracellular proliferation of *Mycobacterium tuberculosis*. PLoS Pathog. 8:e1002691. doi: 10.1371/journal.ppat.1002691, PMID: 22589723 PMC3349752

[ref40] Lamers M. M. Haagmans B. L. (2022). SARS-CoV-2 pathogenesis. Nat. Rev. Microbiol. 20, 270–284. doi: 10.1038/s41579-022-00713-0, PMID: 35354968

[ref41] Li F. (2016). Structure, function, and evolution of coronavirus spike proteins. Annu. Rev. Virol. 3, 237–261. doi: 10.1146/annurev-virology-110615-042301, PMID: 27578435 PMC5457962

[ref42] Li G. De Clercq E. (2020). Therapeutic options for the 2019 novel coronavirus (2019-nCoV). Nat. Rev. Drug Discov. 19, 149–150. doi: 10.1038/d41573-020-00016-0, PMID: 32127666

[ref43] Lim Y. X. Ng Y. L. Tam J. P. Liu D. (2016). Human coronaviruses: a review of virus–host interactions. Diseases 4:26. doi: 10.3390/diseases4030026, PMID: 28933406 PMC5456285

[ref44] Liu D. X. Liang J. Q. Fung T. S. (2021). “Human coronavirus-229E, -OC43, -NL63, and -HKU1 (Coronaviridae)” in Encyclopedia of Virology. eds. Bamford D. Zuckerman M.. Elsevier Ltd., Academic Press, 4th ed, 428–440. doi: 10.1016/B978-0-12-809633-8.21501-X

[ref45] Loo S. L. Wark P. A. B. Esneau C. Nichol K. S. Hsu A. C. Y. Bartlett N. W. (2020). Human coronaviruses 229E and OC43 replicate and induce distinct antiviral responses in differentiated primary human bronchial epithelial cells. Am. J. Physiol. Lung Cell. Mol. Physiol. 319, L926–L931. doi: 10.1152/ajplung.00374.2020, PMID: 32903043 PMC7758816

[ref46] Milewska A. Zarebski M. Nowak P. Stozek K. Potempa J. Pyrc K. (2014). Human coronavirus NL63 utilizes heparan sulfate proteoglycans for attachment to target cells. J. Virol. 88, 13221–13230. doi: 10.1128/JVI.02078-14, PMID: 25187545 PMC4249106

[ref47] Miorin L. Mire C. E. Ranjbar S. Hume A. J. Huang J. Crossland N. A.. (2022). The oral drug nitazoxanide restricts SARS-CoV-2 infection and attenuates disease pathogenesis in Syrian hamsters. bioRxiv. doi: 10.1101/2022.02.08.479634, PMID: 35169796 PMC8845418

[ref48] Morfopoulou S. Brown J. R. Davies E. G. Anderson G. Virasami A. Qasim W.. (2016). Human coronavirus OC43 associated with fatal encephalitis. N. Engl. J. Med. 375, 497–498. doi: 10.1056/NEJMc1509458, PMID: 27518687

[ref49] Ohuchi R. Ohuchi M. Garten W. Klenk H. D. (1997). Oligosaccharides in the stem region maintain the influenza virus hemagglutinin in the metastable form required for fusion activity. J. Virol. 71, 3719–3725. doi: 10.1128/jvi.71.5.3719-3725.1997, PMID: 9094646 PMC191521

[ref50] Pene F. Merlat A. Vabret A. Rozenberg F. Buzyn A. Dreyfus F.. (2003). Coronavirus 229E-related pneumonia in immunocompromised patients. Clin. Infect. Dis. 37, 929–932. doi: 10.1086/377612, PMID: 13130404 PMC7107892

[ref51] Petersen T. Lee Y. J. Osinusi A. Amorosa V. K. Wang C. Kang M.. (2016). Interferon stimulated gene expression in HIV/HCV coinfected patients treated with nitazoxanide/peginterferon-alfa-2a and ribavirin. AIDS Res. Hum. Retrovir. 32, 660–667. doi: 10.1089/aid.2015.0236, PMID: 26974581 PMC4931749

[ref52] Piacentini S. la Frazia S. Riccio A. Pedersen J. Z. Topai A. Nicolotti O.. (2018). Nitazoxanide inhibits paramyxovirus replication by targeting the fusion protein folding: role of glycoprotein-specific thiol oxidoreductase ERp57. Sci. Rep. 8:10425. doi: 10.1038/s41598-018-28172-9, PMID: 29992955 PMC6041319

[ref53] Pizzato Scomazzon S. Riccio A. Santopolo S. Lanzilli G. Coccia M. Rossi A.. (2019). The zinc-finger AN1-type domain 2a gene acts as a regulator of cell survival in human melanoma: role of E3-ligase cIAP2. Mol. Cancer Res. 17, 2444–2456. doi: 10.1158/1541-7786.MCR-19-0243, PMID: 31540997

[ref54] Planas D. Bruel T. Grzelak L. Guivel-Benhassine F. Staropoli I. Porrot F.. (2021). Sensitivity of infectious SARS-CoV-2 B.1.1.7 and B.1.351 variants to neutralizing antibodies. Nat. Med. 27, 917–924. doi: 10.1038/s41591-021-01318-5, PMID: 33772244

[ref55] Pyrc K. Bosch B. J. Berkhout B. Jebbink M. F. Dijkman R. Rottier P.. (2006). Inhibition of human coronavirus NL63 infection at early stages of the replication cycle. Antimicrob. Agents Chemother. 50, 2000–2008. doi: 10.1128/AAC.01598-05, PMID: 16723558 PMC1479111

[ref56] Riccio A. Santopolo S. Rossi A. Piacentini S. Rossignol J. F. Santoro M. G. (2022). Impairment of SARS-CoV-2 spike glycoprotein maturation and fusion activity by nitazoxanide: an effect independent of spike variants emergence. Cell. Mol. Life Sci. 79:227. doi: 10.1007/s00018-022-04246-w, PMID: 35391601 PMC8989121

[ref57] Risku M. Lappalainen S. Räsänen S. Vesikari T. (2010). Detection of human coronaviruses in children with acute gastroenteritis. J. Clin. Virol. 48, 27–30. doi: 10.1016/j.jcv.2010.02.013, PMID: 20233673 PMC7108425

[ref58] Rocco P. R. M. Silva P. L. Cruz F. F. Melo-Junior M. A. C. Tierno P. F. G. M. M. Moura M. A.. (2021). Early use of nitazoxanide in mild Covid-19 disease: randomised, placebo-controlled trial. Eur. Respir. J. 58:2003725. doi: 10.1183/13993003.03725-2020, PMID: 33361100 PMC7758778

[ref59] Rocco P. R. M. Silva P. L. Cruz F. F. Tierno P. F. G. M. M. Rabello E. Junior J. C.. (2022). Nitazoxanide in patients hospitalized with covid-19 pneumonia: a multicentre, randomized, double-blind, placebo-controlled trial. Front. Med. 9:844728. doi: 10.3389/fmed.2022.844728, PMID: 35492335 PMC9043450

[ref60] Rossignol J. F. (2014). Nitazoxanide: a first-in-class broad-spectrum antiviral agent. Antivir. Res. 110, 94–103. doi: 10.1016/j.antiviral.2014.07.014, PMID: 25108173 PMC7113776

[ref61] Rossignol J. F. (2016). Nitazoxanide, a new drug candidate for the treatment of Middle East respiratory syndrome coronavirus. J. Infect. Public Health 9, 227–230. doi: 10.1016/j.jiph.2016.04.001, PMID: 27095301 PMC7102735

[ref62] Rossignol J. F. Abu-Zekry M. Hussein A. Santoro M. G. (2006a). Effect of nitazoxanide for treatment of severe rotavirus diarrhoea: randomised double-blind placebo-controlled trial. Lancet 368, 124–129. doi: 10.1016/S0140-6736(06)68852-1, PMID: 16829296

[ref63] Rossignol J. F. Ayoub A. Ayers M. S. (2001). Treatment of diarrhea caused by Giardia intestinalis and *Entamoeba histolytica* or *E. dispar*: a randomized, double-blind, placebo-controlled study of nitazoxanide. J. Infect. Dis. 184, 381–384. doi: 10.1086/322038, PMID: 11443569

[ref64] Rossignol J. F. Bardin M. C. Fulgencio J. Mogelnicki D. Bréchot C. (2022a). A randomized double-blind placebo-controlled clinical trial of nitazoxanide for treatment of mild or moderate COVID-19. eClinicalMedicine. 45:101310. doi: 10.1016/j.eclinm.2022.101310, PMID: 35237748 PMC8883002

[ref65] Rossignol J. F. Elfert A. el–Gohary Y. Keeffe E. B. (2009a). Improved virologic response in chronic hepatitis C genotype 4 treated with nitazoxanide, peginterferon, and ribavirin. Gastroenterology 136, 856–862. doi: 10.1053/j.gastro.2008.11.037, PMID: 19135998

[ref66] Rossignol J. F. Kabil S. M. el–Gohary Y. Younis A. M. (2006b). Effect of nitazoxanide in diarrhea and enteritis caused by Cryptosporidium species. Clin. Gastroenterol. Hepatol. 4, 320–324. doi: 10.1016/j.cgh.2005.12.020, PMID: 16527695

[ref67] Rossignol J. F. La Frazia S. Chiappa L. Ciucci A. Santoro M. G. (2009b). Thiazolides, a new class of anti-influenza molecules targeting viral hemagglutinin at the post-translational level. J. Biol. Chem. 284, 29798–29808. doi: 10.1074/jbc.M109.029470, PMID: 19638339 PMC2785610

[ref68] Rossignol J. F. Tijsma A. S. L. van Baalen C. A. (2022b). Mechanism of antiviral activity of Nitazoxanide: effect on adenosine triphosphate in influenza-virus infected Madin Darby canine kidney cells. J. Infect. Dis. Preve. Med. 10:262. doi: 10.35841/2329-8731.22.10.262

[ref69] Santopolo S. Riccio A. Rossi A. Santoro M. G. (2021b). The proteostasis guardian HSF1 directs the transcription of its paralog and interactor HSF2 during proteasome dysfunction. Cell. Mol. Life Sci. 78, 1113–1129. doi: 10.1007/s00018-020-03568-x, PMID: 32607595 PMC11071745

[ref70] Santopolo S. Riccio A. Santoro M. G. (2021a). The biogenesis of SARS-CoV-2 spike glycoprotein: multiple targets for host-directed antiviral therapy. Biochem. Biophys. Res. Commun. 538, 80–87. doi: 10.1016/j.bbrc.2020.10.080, PMID: 33303190 PMC7698684

[ref71] Santoro M. G. Amici C. Elia G. Benedetto A. Garaci E. (1989). Inhibition of virus protein glycosylation as the mechanism of the antiviral action of prostaglandin A in Sendai virus-infected cells. J. Gen. Virol. 70, 789–800. doi: 10.1099/0022-1317-70-4-789, PMID: 2543761

[ref72] Santoro M. G. Carafoli E. (2020). Remdesivir: from Ebola to COVID-19. Biochem. Biophys. Res. Commun. 538, 145–150. doi: 10.1016/j.bbrc.2020.11.043 33388129 PMC7836944

[ref73] Santoro M. G. Ciucci A. Gianferretti P. Belardo G. La Frazia S. Carta S.. (2007). Thiazolides: a new class of broad-spectrum antiviral drugs targeting virus maturation. Antivir. Res. 74:A31. doi: 10.1016/j.antiviral.2007.01.019

[ref74] Santoro M. G. Favalli C. Mastino A. Jaffe B. M. Esteban M. Garaci E. (1988). Antiviral activity of a synthetic analog of prostaglandin A in mice infected with influenza A virus. Arch. Virol. 99, 89–100. doi: 10.1007/BF01311026, PMID: 3355375

[ref75] Santoro M. G. Jaffe B. M. Garaci E. Esteban M. (1982). Antiviral effect of prostaglandins of the A series: inhibition of vaccinia virus replication in cultured cells. J. Gen. Virol. 63, 435–440. doi: 10.1099/0022-1317-63-2-435, PMID: 6185627

[ref76] Shirato K. Imada Y. Kawase M. Nakagaki K. Matsuyama S. Taguchi F. (2014). Possible involvement of infection with human coronavirus 229E, but not NL63, in Kawasaki disease. J. Med. Virol. 86, 2146–2153. doi: 10.1002/jmv.23950, PMID: 24760654 PMC7166330

[ref77] Shou J. Wang M. Cheng X. Wang X. Zhang L. Liu Y.. (2020). Tizoxanide induces autophagy by inhibiting PI3K/Akt/mTOR pathway in RAW264.7 macrophage cells. Arch. Pharm. Res. 43, 257–270. doi: 10.1007/s12272-019-01202-4, PMID: 31894502

[ref78] Sicari D. Chatziioannou A. Koutsandreas T. Sitia R. Chevet E. (2020). Role of the early secretory pathway in SARS-CoV-2 infection. J. Cell Biol. 219:e202006005. doi: 10.1083/jcb.202006005, PMID: 32725137 PMC7480111

[ref79] Silva M. Espejo A. Pereyra M. L. Lynch M. Thompson M. Taconelli H.. (2021). Efficacy of Nitazoxanide in reducing the viral load in COVID-19 patients. Randomized, placebo-controlled, single-blinded, parallel group, pilot study. medRxiv. doi: 10.1101/2021.03.03.21252509

[ref80] Sokhela S. Bosch B. Hill A. Simmons B. Woods J. Johnstone H.. (2022). Randomized clinical trial of nitazoxanide or sofosbuvir/daclatasvir for the prevention of SARS-CoV-2 infection. J. Antimicrob. Chemother. 77, 2706–2712. doi: 10.1093/jac/dkac266, PMID: 35953881 PMC9384711

[ref81] Son J. Huang S. Zeng Q. Bricker T. L. Case J. B. Zhou J.. (2022). JIB-04 has broad-spectrum antiviral activity and inhibits d3SARS-CoV-2 replication and coronavirus pathogenesis. MBio 13:e0337721. doi: 10.1128/mbio.03377-21, PMID: 35038906 PMC8764536

[ref82] Stachulski A. V. Rossignol J. F. Pate S. Taujanskas J. Robertson C. M. Aerts R.. (2021). Synthesis, antiviral activity, preliminary pharmacokinetics and structural parameters of thiazolide amine salts. Future Med. Chem. 13, 1731–1741. doi: 10.4155/fmc-2021-0055, PMID: 34402654

[ref83] Stevens L. J. Pruijssers A. J. Lee H. W. Gordon C. J. Tchesnokov E. P. Gribble J.. (2022). Mutations in the SARS-CoV-2 RNA-dependent RNA polymerase confer resistance to remdesivir by distinct mechanisms. Sci. Transl. Med. 14:eabo0718. doi: 10.1126/scitranslmed.abo0718, PMID: 35482820 PMC9097878

[ref84] Tang X. Wu C. Li X. Song Y. Yao X. Wu X.. (2020). On the origin and continuing evolution of SARS-CoV-2. Natl. Sci. Rev. 7, 1012–1023. doi: 10.1093/nsr/nwaa036, PMID: 34676127 PMC7107875

[ref85] Trabattoni D. Gnudi F. Ibba S. V. Saulle I. Agostini S. Masetti M.. (2016). Thiazolides elicit anti-viral innate immunity and reduce HIV replication. Sci. Rep. 6:27148. doi: 10.1038/srep27148, PMID: 27250526 PMC4890011

[ref86] Tyrrell D. A. J. Bynoe M. L. (1965). Cultivation of a novel type of common cold virus in organ culture. Br. Med. J. 1, 1467–1470. doi: 10.1136/bmj.1.5448.1467, PMID: 14288084 PMC2166670

[ref87] van der Hoek L. Pyrc K. Jebbink M. F. Vermeulen-Oost W. Berkhout R. J. M. Wolthers K. C.. (2004). Identification of a new human coronavirus. Nat. Med. 10, 368–373. doi: 10.1038/nm1024, PMID: 15034574 PMC7095789

[ref88] Vijgen L. Keyaerts E. Moës E. Maes P. Duson G. van Ranst M. (2005). Development of one-step, real-time, quantitative reverse transcriptase PCR assays for absolute quantitation of human coronaviruses OC43 and 229E. J. Clin. Microbiol. 43, 5452–5456. doi: 10.1128/JCM.43.11.5452-5456.2005, PMID: 16272469 PMC1287813

[ref89] Wahl A. Gralinski L. E. Johnson C. E. Yao W. Kovarova M. Dinnon K. H. III. (2021). SARS-CoV-2 infection is effectively treated and prevented by EIDD-2801. Nature 591, 451–457. doi: 10.1038/s41586-021-03312-w, PMID: 33561864 PMC7979515

[ref90] Wang M. Cao R. Zhang L. Yang X. Liu J. Xu M.. (2020). Remdesivir and chloroquine effectively inhibit the recently emerged novel coronavirus (2019-nCoV) in vitro. Cell Res. 30, 269–271. doi: 10.1038/s41422-020-0282-0, PMID: 32020029 PMC7054408

[ref91] Wang X. Shen C. Liu Z. Peng F. Chen X. Yang G.. (2018). Nitazoxanide, an antiprotozoal drug, inhibits late-stage autophagy and promotes ING1-induced cell cycle arrest in glioblastoma. Cell Death Dis. 9:1032. doi: 10.1038/s41419-018-1058-z, PMID: 30302016 PMC6177448

[ref92] Wen W. Chen C. Tang J. Wang C. Zhou M. Cheng Y.. (2022). Efficacy and safety of three new oral antiviral treatment (molnupiravir, fluvoxamine and Paxlovid) for COVID-19: a meta-analysis. Ann. Med. 54, 516–523. doi: 10.1080/07853890.2022.2034936, PMID: 35118917 PMC8820829

[ref93] Woo P. C. Y. Lau S. K. P. Chu C. Chan K. H. Tsoi H. W. Huang Y.. (2005). Characterization and complete genome sequence of a novel coronavirus, coronavirus HKU1, from patients with pneumonia. J. Virol. 79, 884–895. doi: 10.1128/JVI.79.2.884-895.2005, PMID: 15613317 PMC538593

[ref94] Xu C. Ng D. T. W. (2015). Glycosylation-directed quality control of protein folding. Nat. Rev. Mol. Cell Biol. 16, 742–752. doi: 10.1038/nrm4073, PMID: 26465718

[ref95] Zhang Z. Liu W. Zhang S. Wei P. Zhang L. Chen D.. (2022). Two novel human coronavirus OC43 genotypes circulating in hospitalized children with pneumonia in China. Emerg. Microbes Infect. 11, 168–171. doi: 10.1080/22221751.2021.2019560, PMID: 34907853 PMC8741245

