# Supplementary material for: The FDA-approved drug nitazoxanide is a potent inhibitor of human seasonal coronaviruses acting at postentry level: effect on the viral spike glycoprotein
Source: Front Microbiol. 2023 Aug 29;14:1206951. doi: 10.3389/fmicb.2023.1206951 (PMC10497118; doi:10.3389/fmicb.2023.1206951)
Supplement: Supplementary file 1 [file Data_Sheet_1.PDF]

Supplementary Information

The FDA-approved drug nitazoxanide is a potent inhibitor of human seasonal coronaviruses acting at postentry level: effect on the viral spike glycoprotein

Sara Piacentini[1#], Anna Riccio[1#], Silvia Santopolo[1], Silvia Pauciullo[1], Simone La Frazia[1], Antonio Rossi[2], Jean-Francois Rossignol[3] and M. Gabriella Santoro[1,2*]

[1]Department of Biology, University of Rome Tor Vergata, Rome, Italy; [2]Institute of Translational Pharmacology, CNR, Rome, Italy; [3]Romark Institute of Medical Research, Tampa, Florida, USA

Supplementary Figure Legends

Supplementary Figure 1. Antiviral activity of nitazoxanide in human lung cells at different times after infection with OC43 and 229E HCoVs.

(**A,B**) MRC-5 cells mock-infected or infected with HCoV-229E (A) or HCoV-OC43 (B) at an MOI of 0.1 $TCID_{50}$/cell were treated with 1 μg/ml NTZ (filled bars), or vehicle (empty bars) immediately after the adsorption period. Extracellular viral RNA levels were determined in the cell supernatants at 48h after infection by qRT-PCR. (**C,D**) MRC-5 cells mock-infected or infected with HCoV-229E (C) or HCoV-OC43 (D) at an MOI of 0.5 $TCID_{50}$/cell were treated as in (A,B). Extracellular viral RNA levels were determined at 24h p.i. by qRT-PCR. (**A-D**) Data, expressed as percent of untreated control, represent the mean ± S.D. of duplicate samples. *= $p< 0.01$; Student's *t*-test.

Supplementary Figure 2. Effect of nitazoxanide treatment started at different times after infection with HCoV-229E in human lung cells.

MRC-5 cells mock infected or infected with HCoV-229E at an MOI of 0.5 $TCID_{50}$/cell were treated with NTZ (1 μg/ml) or vehicle at the indicated times after virus adsorption. Virus yields were determined at 24h p.i. by infectivity assay. Data, expressed as $TCID_{50}$/ml, represent the mean ± S.D. of duplicate samples. *= $p< 0.01$; Student's *t*-test.

Supplementary Figure 3. Nitazoxanide does not inhibit HCoV-229E spike protein expression in MRC-5 lung cells.

Confocal images of HCoV-229E spike glycoprotein (red) in MRC-5 cells mock-infected or infected with HCoV-229E at an MOI of 0.5 $TCID_{50}$/cell and treated with NTZ (1 μg/ml) or vehicle for 24h. Nuclei are stained with Hoechst (blue). Merge images are shown. Scale bar, 20 μm.

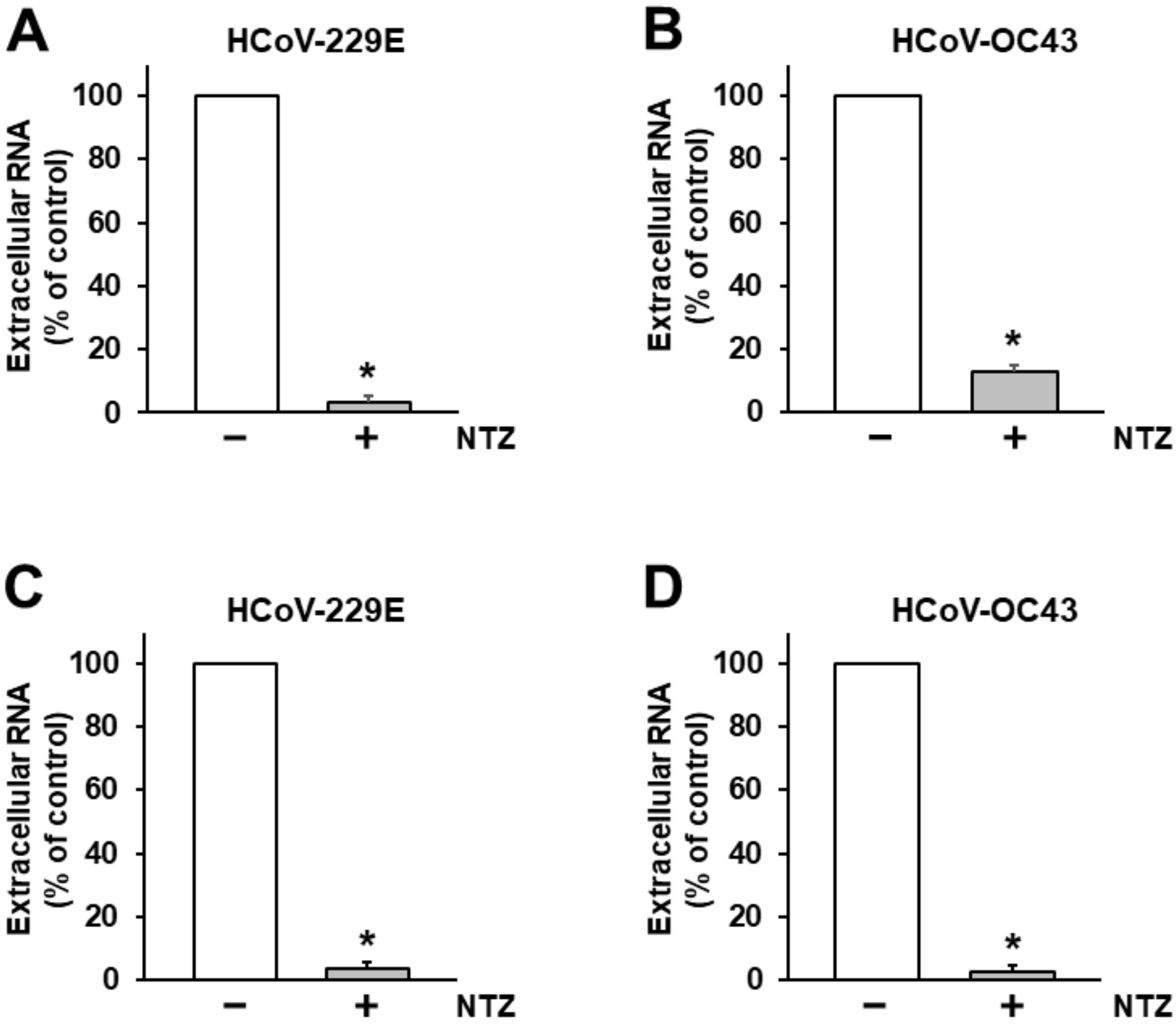
A
HCoV-229E
Extracellular RNA (% of control)
100
80
60
40
20
0
*
−
+
NTZ
B
HCoV-OC43
Extracellular RNA (% of control)
100
80
60
40
20
0
*
−
+
NTZ
C
HCoV-229E
Extracellular RNA (% of control)
100
80
60
40
20
0
*
−
+
NTZ
D
HCoV-OC43
Extracellular RNA (% of control)
100
80
60
40
20
0
*
−
+
NTZ

Figure S1

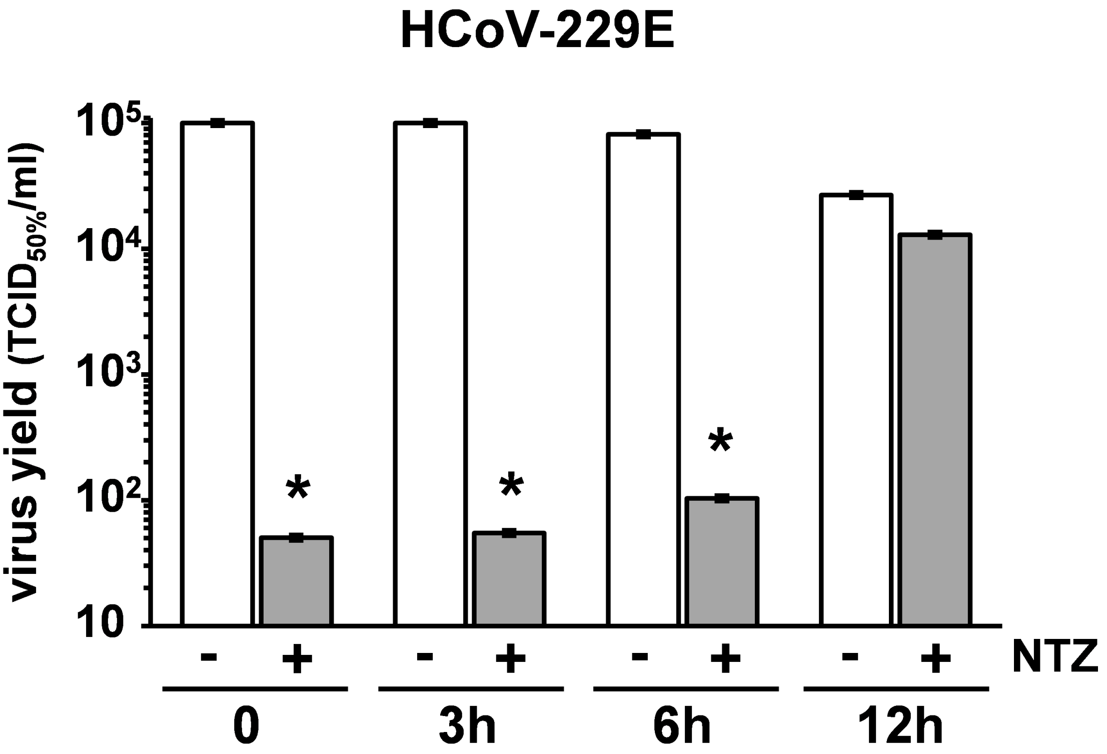
HCoV-229E
virus yield (TCID50%/ml)
10^5
10^4
10^3
10^2
10
*
*
*
- + - + - + - + NTZ
0
3h
6h
12h

Figure S2

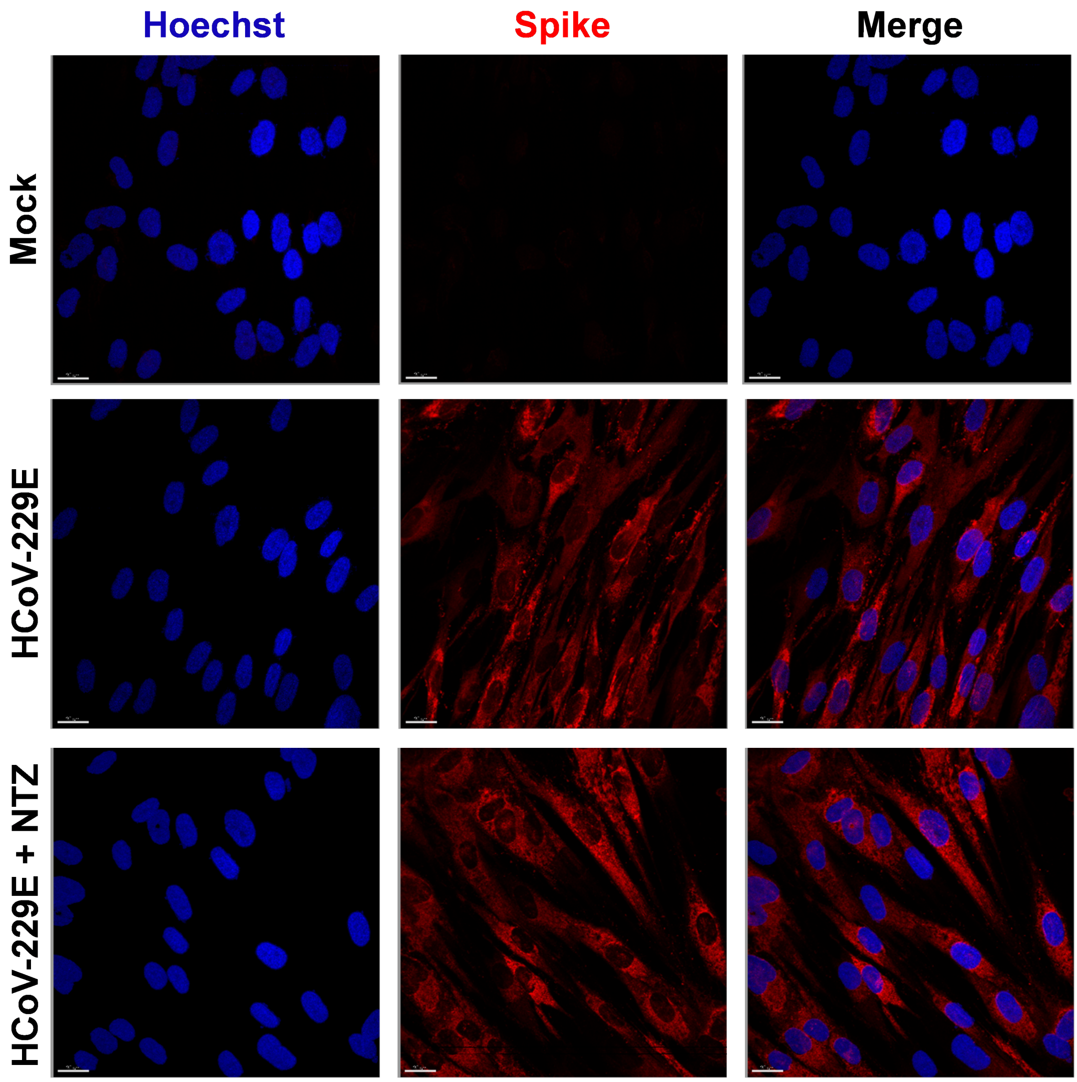

Figure S3
